# Neuroadaptive Bayesian optimisation to study individual differences in infants’ engagement with social cues

**DOI:** 10.1016/j.dcn.2024.101401

**Published:** 2024-06-10

**Authors:** A. Gui, E. Throm, P.F. da Costa, F. Penza, M. Aguiló Mayans, A. Jordan-Barros, R. Haartsen, R. Leech, E.J.H. Jones

**Affiliations:** a Centre for Brain and Cognitive Development, Department of Psychological Science, Birkbeck, University of London, Malet Street, London WC1E 7HX, United Kingdom; b Department of Psychology, University of Essex, Wivenhoe Park, Colchester CO4 3SQ, United Kingdom; c Department of Neuroimaging, Institute of Psychiatry, Psychology and, Neuroscience, King’s College London, de Crespigny Road, London SE5 8AB, United Kingdom

**Keywords:** Infant, Socialisation, Individualised electro-encephalography, Gaze, Emotion, Neuroadaptive Bayesian optimisation

## Abstract

Infants’ motivation to engage with the social world depends on the interplay between individual brain’s characteristics and previous exposure to social cues such as the parent’s smile or eye contact. Different hypotheses about why specific combinations of emotional expressions and gaze direction engage children have been tested with group-level approaches rather than focusing on individual differences in the social brain development. Here, a novel Artificial Intelligence-enhanced brain-imaging approach, Neuroadaptive Bayesian Optimisation (NBO), was applied to infant electro-encephalography (EEG) to understand how selected neural signals encode social cues in individual infants. EEG data from 42 6- to 9-month-old infants looking at images of their parent’s face were analysed in real-time and used by a Bayesian Optimisation algorithm to identify which combination of the parent’s gaze/head direction and emotional expression produces the strongest brain activation in the child. This individualised approach supported the theory that the infant’s brain is maximally engaged by communicative cues with a negative valence (angry faces with direct gaze). Infants attending preferentially to faces with direct gaze had increased positive affectivity and decreased negative affectivity. This work confirmed that infants’ attentional preferences for social cues are heterogeneous and shows the NBO's potential to study diversity in neurodevelopmental trajectories.

## 1 Introduction

“Why are we social?” is a question that philosophers and psychologists started to investigate more than 2000 years ago (cft. Aristotle, Politics, 1.1253.a). Social affiliation begins very early in infancy, with the development of a network in the brain that becomes specialised to process the complex social and communicative skills that characterise our species (Grossmann and Johnson, 2007, Mundy and Newell, 2009). This network of brain regions contributing to social cognition emerges during the first year of life (Johnson, 2011) and is reinforced and refined based on social information to which the individual is exposed (Klein et al., 2009).

The developmental mechanisms that shape the social brain are shared by all human beings but expressed in a way that is unique to each individual, as their intrinsic characteristics and environmental exposure both play a role in shaping developmental trajectories (Shultz et al., 2018). Theories of social brain development agree that at birth the brain responds to a few stimuli, including face-like patterns and eye gaze, due to their sensory properties (Johnson, 2011, Shultz et al., 2018, Simion et al., 2002). Over the following months, a network of brain regions that are activated by such highly salient stimuli is fine-tuned based on experience and becomes less responsive to other, less engaging stimuli (Johnson, 2001, Johnson et al., 2005). The combination of which cues elicit attention in all infants on the one hand, and how attention engagement is increasingly modified by what the individual infant has experienced on the other hand, will gate social learning and motivate the human being to engage in social interaction from a very early age.

To understand the origins of humans’ interest in the social world it is therefore important to identify what social cues are most engaging for infants when their social brain network is starting to specialise, that is in the second half of the first year of life (Jones et al., 2015). Indeed, by six months of age infants are already unique in what experiences with social stimuli they had and in the way they learnt to process them. For example, the first six months of age is when infants have the most extensive exposure to their caregiver and other familiar adults’ faces (Jayaraman et al., 2017), which offer experience with highly salient social cues such as facial expressions and gaze direction (Carnevali et al., 2022). Additionally, it has been proposed that infants’ and parents’ social cues iteratively adapt to each other in the very first months, such that the infants’ own responses might reinforce or modify parents’ cues, shaping their own exposure to social stimuli (Shultz et al., 2018). Indeed, neural correlates of social attention engagement at the end of the first year of life are associated with later cognitive skills (Jones et al., 2020), supporting the idea that attention engagement to social cues is key for early and effective learning (Çetinçelik et al., 2021, Colombo et al., 2004, Csibra and Gergely, 2009a, Csibra and Gergely, 2009b, Csibra and Gergely, 2011). Thus, intrinsic and experience-based individual differences in attention engagement to social cues are likely to contribute to the tuning of the social brain in the second half of the first year, and have cascading effects on the infant’s cognitive development.

### 1.1 Two engaging social cues: Gaze and emotion

Gaze direction is a critical social cue that contains information about the direction of attention of a communicative partner. The ability to discern differences in gaze direction begins from birth: seminal research showed that from birth infants look more at faces with a direct gaze than faces with averted gaze (Farroni et al., 2000, Farroni et al., 2002, Farroni et al., 2006). Newborns and 4-month-old infants are more likely to recognise the identity of faces with direct gaze, suggesting that direct gaze can potentiate memory or focus attention (Rigato et al., 2011). By the second half of the first year, infants know that gaze and head turn towards an object are communicative signals intending to direct their attention away from the face and towards the cued direction (Del Bianco et al., 2018). Further, direct gaze preceding a gaze shift and head turn facilitates 6- and 9-months old infants in orienting towards the gazed-at toy compared to when these cues are absent (Senju and Csibra, 2008, Senju et al., 2008, Szufnarowska et al., 2014). Importantly, Pons et al., (2004) found that infants who looked more to their mothers’ eyes at 6 months manifested higher social and communication skills at later ages. This indicates on the one hand that early eye contact might have a scaffolding role for later socialisation, and on the other hand that individual differences might overlay general group-level effects at a young age.

Facial expressions also provide information about the intent and mood of a communicative partner. From the third month of age, infants are particularly attracted to faces displaying happy facial expressions (Turati et al., 2011, Brenna et al., 2013). Faces displaying emotions (e.g. happiness, anger) facilitate rule learning (Quadrelli et al., 2020) and face recognition (Gross and Schwarzer, 2010, Schwarzer and Jovanovic, 2010) in 7- to 8-month-old infants, suggesting they elicit greater attention. Indeed, 7- to 8-month-old infants tend to look longer at happy and angry faces than sad ones (Kim and Johnson, 2013, Yong and Ruffman, 2016). Research also shows that in infants who are younger than one year of age, the degree of attentional engagement with angry (but not happy) faces depends on the infants’ levels of negative affect (Pérez-Edgar et al., 2017), suggesting that individual variability in temperamental characteristics is linked to neurodevelopment. Taken together, these studies show that expressive faces may elicit deeper levels of attention. However, group effects are overlaid with individual differences between infants such that the distribution of attention across emotions may depend on the emotional traits of the infant and their experiences. Both elements shape the tuning of the individual infants’ social brain network and subsequent processing of social cues such as emotional expressions.

In the real world, cues are experienced together when viewing faces and there is evidence that infants integrate gaze and head direction and emotional expression from early on. For example, at four months of age infants can orient in the direction of a gaze shift, but faces displaying happy or fearful emotional expressions seem to hold their attention and reduce speed to orient towards the gazed-at object (Rigato et al., 2013). Between 9 and 12 months of age, infants are faster at following a gaze cue when a model displays a happy versus angry or fearful face (Niedźwiecka and Tomalski, 2015), suggesting that over the second half of the first year of life infants show a developmental reversal for happy faces. Similarly, 10-month-old infants are faster to orient towards a peripheral object when a happy face with direct gaze is displayed on the centre of the screen at the same time, while it takes them longer to disengage from the central facial stimulus when the face displays anger, independent of gaze direction (Doi et al., 2010). Thus, emotional expression can influence the child’s speed of reaction to gaze. Both gaze direction and emotional expression are powerful social cues that capture infants’ attention and play a role in learning. The degree of attention engagement to these social cues is linked with the infant’s temperamental characteristics and their *combined* effect on infant social attention could be an indicator of later social development.

There is broad consensus in the literature that emotional expression and gaze direction play a role in capturing infants’ attention towards faces. This developmental process has been formalised by the Natural Pedagogy framework (Csibra and Gergely, 2009a, Csibra and Gergely, 2009b). According to this theory, infants are most attentive to ostensive cues that indicate that they are being addressed, as they understand a message is about to be conveyed to them. In this framework, infants would show the strongest attentional response to happy expression accompanied by direct gaze, reflecting the other’s intention to communicate with them, compared to neutral or angry expressions. However, different theoretical explanations emerging from adult ERP studies have been used to explain the infant Nc results that are not consistent with the Natural Pedagogy theory.

An alternative account is the Negativity Bias hypothesis, suggesting that infants respond more rapidly to facial cues when they are biologically important for them to be detected, such as angry expressions directed toward the individual (Klucharev and Sams, 2004). Following this account, one would expect a stronger attentional response to an angry expression accompanied by direct gaze compared to a happy expression accompanied by direct gaze or to an angry expression accompanied by averted gaze.

A third account is represented by the Shared Signal hypothesis, which proposes that infants pay attention to specific combinations of gaze direction and emotional expression that facilitate approach or avoidance behaviours (Rigato et al., 2010). In particular, it suggests that the perception of a happy and angry expression is more enhanced when accompanied by a direct gaze (compared to averted gaze), facilitating approaching behaviour, while the perception of fearful or sad expressions is more enhanced when accompanied by averted gaze (compared to direct gaze), facilitating avoiding behaviour (Adams and Kleck, 2005).

These theories offer different predictions of what characteristics of a face draw infants’ attention and the reasons why young children are motivated towards these social cues in the first instance. However, they do not explain how the proposed general developmental processes intersect with individual differences, e.g., whether the response to social cues varies across infants as a function of their temperamental and behavioural characteristics, and whether these interact with experience. It has been proposed that social development depends on the parent-infant mutually-reinforcing experiences with social cues, such that infants’ smiling faces might induce parents to respond with a smiling face as well, while infants’ distress and difficulties in adapting to social contexts might induce avoidance responses in the parent (Shultz et al., 2018). In this framework, preferential attention engagement to faces’ emotional expressions and gaze direction might be linked to infant’s characteristics and experiences. For example, infants with reduced social skills, those who show more distress or smile less in response of parent’s attempt to interact, and those whose parents are smiling less or looking less at them during everyday interactions might be less exposed to happy faces with a direct gaze and show preferential engagement for non-happy faces with averted gaze and head (Pons et al., 2019). Mapping how the individual infant’s brain would respond to the (virtually infinite) combination of social cues it is exposed to will be crucial to understand what triggers the development of the social brain network, and how it changes based on individual differences.

### 1.2 A neural signature of social attention engagement in infants

One of the most established methods to research infant social attention engagement is electro-encephalography (EEG). This non-invasive neuroimaging technique uses sensors placed on the infant’s head to record changes in the electrical field at the scalp level and infer changes activity of the underlying cortex (Michel et al., 2009). Event-related potentials (ERPs) are changes in electrical brain activity time-locked to events, such as a presentation of a visual stimulus. ERPs are obtained by averaging the time-locked brain activity across multiple trials and pre-defined regions of the scalp, depending on the ERP component of interest (Luck, 2014). These neural signatures have been widely used to study attentional processes in the infant brain (Richards et al., 2010).

The Nc is a negative ERP component typically measured between 250 and 800 ms after the stimulus presentation over the fronto-central electrodes (Courchesne et al., 1981, de Haan et al., 2003). Studies combining EEG with heart-rate and eye-tracking measurements demonstrated that the Nc amplitude is larger (i.e., more negative) during physiologically defined periods of attention regardless of stimulus type (Richards, 2003, Reynolds and Richards, 2005, Reynolds et al., 2010, Guy et al., 2016). For example, Conte et al., (2020) showed that this component is enhanced in 6- to 8-month-old infants during states of heart-rate defined attention. They found that the Nc is stronger during attention to faces than to objects, indicating that infants at this age might be more actively engaged when watching images of faces. Further, typically developing infants present a more enhanced Nc in response to the mother’s face, compared to a non-familiar face (de Haan and Nelson, 1997, Luyster et al., 2014). Thus, the Nc can be used to index the capture of attention by different facial cues.

The Nc is furthermore sensitive to emotional expression. Studies on the Nc amplitude in response to different facial expressions (accompanied by direct gaze) in the first year of life have produced mixed results, with some of them showing that happy faces elicited a larger Nc and others finding fearful or angry faces as producing an enhanced engagement. Nelson and de Haan (1996) found a stronger peak amplitude of the Nc to happy than fearful faces, while no difference between angry and fearful faces was found in 7-month-old infants. van den Boomen et al., 2019a, van den Boomen et al., 2019b found that happy faces elicited a stronger Nc mean amplitude than neutral faces at 9–10 months. In contrast, Xie et al., (2019) found that Nc mean amplitude was larger for angry than for fearful and happy faces, with no change of such effect between 5 and 12 months of age. Similarly, Kobiella et al. (2008) observed a stronger Nc for angry compared to fearful faces in 7-month-old infants. These inconsistent findings might reveal that this neural signature of preferential attention engagement to emotional faces varies as a result of individual differences in temperament and experience. Indeed, infant temperament and parental positive affect seem to be related to Nc peak amplitude in response to emotional faces, such that highly positive 7-month-old children, as recorded with the Smiling-and-Laughter subscale of the Infant Behavior Questionnaire (IBQ; Garstein and Rothbart, 2003; Rothbart, 1981) of positive mothers (whose general mood was measured with the Positive and Negative Affect Schedule, PANAS, Watson et al., 1988) show enhanced Nc in response to fearful vs happy faces (de Haan et al., 2004).

The Nc is also somewhat sensitive to gaze direction. For example, in 5-month-olds Nc mean amplitude is larger for neutral faces with direct than averted gaze (Parise and Csibra, 2013). We also recently observed that typically developing infants aged between 6 and 10 months showed overall larger Nc mean amplitude when looking at faces with a direct gaze compared with averted gaze, but there was inter-individual variability in this response, such that results at the group level were not statistically significant (Gui et al., 2021a, Gui et al., 2021b). Exploratory analyses revealed that typically developing infants with higher scores in the Distress to limitations subscale of the Infant Behavior Questionnaire – Revised (Garstein and Rothbart, 2003) reflecting distress during caretaking activities, confinement and inability to perform desired action, showed enhanced Nc mean amplitude to faces with an averted vs direct gaze (reported in the Supplementary Materials, Figure S1). Moreover, infants’ positive affect in the interaction with the parent is linked to neural sensitivity to gaze direction measured as the latency of the P400, a positive ERP component arguably generated by dipole sources that produce the Nc (Guy et al., 2016). These findings are in line with the Natural Pedagogy theory, suggesting that infants are highly engaged by faces that show communicative intentions, and the strength of the neural response may depend on individual differences in the infant’s characteristics or experience in social context.

Previous EEG studies used the Nc as a signature of infant attention engagement to investigate how the effects of gaze direction interact with emotion. Some findings support the Natural Pedagogy theory (Csibra and Gergely, 2009a, Csibra and Gergely, 2009b), predicting strongest attention engagement when the cues are creating a sense of being addressed in the infant, such as happy faces accompanied by direct gaze. In 4-month-olds, Rigato et al., (2010) found that the Nc amplitude was larger in response to happy versus fearful faces in the presence of direct but not averted gaze. In another study, 7-month-olds did not show a differential Nc response for fearful vs neutral faces accompanied by direct gaze (Hoehl et al., 2008a).

In line with the Negativity Bias hypothesis, a study with 3-month-olds showed a more negative Nc in response to angry expressions when these were accompanied by direct gaze, compared to angry faces accompanied by averted gaze, and to direct gaze with happy and fearful expressions (Hoehl et al., 2008b). In a different study, 7-month-old infants showed a stronger Nc for angry faces accompanied by direct gaze compared to averted gaze, while this difference was not observed with fearful faces (Hoehl and Striano, 2008).

Most of these findings equally support the Shared Signal hypothesis predicting greater attention engagement for happy or angry faces accompanied by direct gaze, or fearful or sad faces accompanied by averted gaze. However, some findings are inconsistent with this theory. For example, neither 4-month-old (Rigato et al., 2010) nor 7-month-old infants (Hoehl et al., 2008a) showed stronger attention engagement for fearful compared to happy or neutral faces, respectively, when accompanied by averted gaze, in contrast to the predictions of the Shared Signal hypothesis.

In sum, these findings indicate that the specific combinations of gaze direction and expression capture infants’ attention differently. Currently, findings as to how exactly these two powerful cues jointly affect infant attention remain mixed and cannot be fully explained by neither of the frameworks at hand. Indeed, the response might be different in different infants, as revealed by some inconsistent findings (van den Boomen et al., 2019a, van den Boomen et al., 2019b and large inter-individual variability in group-level results (Gui et al., 2021a, Gui et al., 2021b). Hence, one important source of variability may be individual differences in infant behaviour, such that infants who have more experience with happy faces with a direct gaze show higher attention engagement to communicative faces at a neural level. On the contrary, faces with an averted gaze and head might be more engaging to infants who are typically distressed in social contexts. Further, smiling faces with direct gaze might be more engaging than more serious (neutral/angry) faces, particularly for children who have more experience with happy, communicative faces as their parents have a positive mood. Further, parents with neutral facial expressions are even expected to be considered hostile by typical infants, as suggested by behavioural responses of distress in the ‘still face’ paradigm (Tronick et al., 1978). Here, we propose a novel approach based on individualised neuroimaging from adult research to create individual-level insights that can test the theoretical frameworks that are most supported by traditional group-level and individual-level approaches. Further, we aim to explore whether individual differences derived from a combination between intrinsic processing types and experience, can account for differences in neural engagement with combined social cues.

### 1.3 Neuroadaptive Bayesian Optimisation to study social attention engagement

The traditional experimental approach used in the studies described above tests the responses of a group of infants to a few pre-selected stimuli (e.g., to an angry vs happy face accompanied by either direct or averted gaze resulting in 2–4 stimuli). However, daily social experiences offer a much larger range of shades between these extreme conditions, for example a range of more or less nuanced facial expressions or gaze and head directions. Moreover, in real life infants experience social cues in integrative ways. For example, the infant’s mother and father will make eye contact when smiling at their baby child, offering both gaze/head direction and emotional expression information simultaneously. Thus, to understand the origins of socialisation we need to study how the individual infant’s brain integrates these cues, and whether differences in neural activation are associated to differences in behavioural characteristics or experiences. However, accounting for the richness in stimuli the child encounters in real-life would require the collection of EEG data in response to a large number of stimuli, which is not a feasible approach due to infants’ short attention span.

In traditional infant designs, stimuli and/or the composition of the study sample are manipulated in order to optimally capture differences linked to the manipulation. Statistical inference approaches test whether stimuli manipulations produce mean differences in brain responses averaged across infants, effectively cancelling out individual differences in favour of group/condition differences. Importantly, *a priori* selected stimuli might not be equally engaging for all the children. However, classic ERP designs are not optimised to study these individual differences in attention engagement. Crucially, these differences in stimulus preference and compliance to the task might be related to individual differences in cognitive and behavioural characteristics, providing a valuable source of insight about individual trajectories of early social development. Thus, while this classic ERP approach has revealed important insights about early social development, including those reported above, it is limited in extent to which it is able to study individual differences in attending and integrating social cues such as gaze/head direction and emotional expressions.

**Fig. 1:**
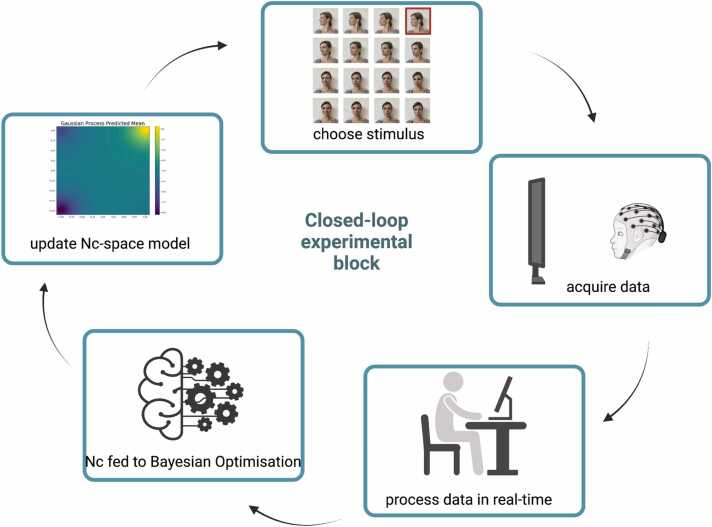
Iterative steps of the closed-loop experiment, which repeat for each block until the optimum is identified. Created with BioRender.com.

A novel method integrating real-time data collection and machine learning techniques has shown promise in adult research to overcome these limitations (Lorenz et al., 2016, Lorenz et al., 2018). This method, called Neuroadaptive Bayesian Optimisation (NBO), is based on a Bayesian Optimisation algorithm that sequentially selects experimental conditions based on real time analysis of a selected brain response (Lorenz et al., 2017). This process is repeated as the algorithm obtains neural metrics for a definite number of stimuli organised within an experimental ‘search space’, including a potentially infinite combination of stimuli characteristics. Obtaining feedback about the estimated brain response to the presented stimuli in real time, the algorithm can make predictions about the location of the search space that maximises the information about the infant’s brain response, corresponding to the stimulus that produces the strongest activation, without having to sample all the possible stimuli in the space (Fig. 1). This is possible because the search algorithm interpolates between the sampled data points using Gaussian Processes. These models further allow access to information regarding the uncertainty of a given prediction by using the Gaussian variance as a proxy for uncertainty.

We recently argued that using NBO in infant research is a promising avenue to produce reliable and meaningful findings (da Costa et al., 2021, Gui et al., 2022) and we now use this approach to ask a theoretical question. First, the machine learning algorithm acts to efficiently work out which is the stimulus producing the strongest brain activation. The algorithm aims to reach the optimal stimulus and stops the paradigm as soon as a solution is found, avoiding overly long paradigms that produce data that are no longer informative. Second, in this individualised approach the algorithm selects the next stimulus to be presented to the infants based on their brain responses which do not depend on group differences. Thus, the paradigm is necessarily personalised and aims to produce maximal engagement in the child. Third, since the entire EEG data processing pipeline needs to be pre-defined a priori as data will need to be cleaned and an output neural metric produced in real-time after each block, the protocol itself represents a pre-registration of the proposed approach which cannot be modified post-hoc.

For the NBO approach to work, we need a reliable signal that can be used as a correlate of attention engagement towards social stimuli. The Nc is the perfect candidate for this purpose, having been validated by such a long history of research as a correlate of infant attention engagement with social cues between 6 and 9 months of age (Conte et al., 2020). Furthermore, the Nc mean amplitude to faces has proved to be a neural signature with moderate test-retest reliability, with interclass correlation between Nc mean amplitude collected in two subsequent visits of the same infant = 0.57 (Munsters et al., 2019a). Of note, the Nc peak amplitude recorded in infants aged between 4.5 and 7.5 months is also sensitive to novelty (Richards et al., 2010). In the present study, we used the parent’s face as stimulus rather than an unknown model’s face; this way we avoided proposed target measure, the amplitude of the negative ERP around the Nc peak, to be confounded by familiarity with the face or similarity of the model’s face to the parent’s face (de Haan and Nelson, 1997). Our personalised approach presented a range of emotional expressions and gaze/head directions of the parent’s face, in order to evaluate the intersection between general accounts for preferential attention engagement and individual differences based on infants’ characteristics and experience.

### 1.4 The present study

In the present study, which was in-principle accepted as a registered report, we aimed to apply NBO to infant EEG to understand what combination of the parent’s gaze/head direction and emotional expression is more engaging for 6- to 9-month-old infants and thereby produce insights at both group and individual level. Since at this age the mother’s face has been shown to elicit an enhanced Nc compared to a stranger’s face (de Haan and Nelson, 1997, de Haan and Nelson, 1999), the possible experimental stimuli consisted in a range of pictures of the parent’s face displaying different emotional expressions and directed toward or away from the child. Combinations of the parents’ faces displaying a range of emotional expressions and gaze/head directions were arranged in a 4 by 4 ‘search space’ (Figure S1). Faces varied along the *Emotion* dimension (on the x axis) in four steps from very happy with a large smile, to happy with a small smile, to neutral to angry, and along the *Gaze* dimension (on the y axis) from direct gaze to head turned away horizontally by 90 degrees in 4 steps. Although in the past infants as young as 5 months were shown to decrease attention for faces with a gaze shifted away horizontally by approximately 5 degrees (Symons et al., 1998), there is also evidence that children as old as 5 years detect a gaze as averted only when it is shifted by on average 15 degrees or more, with this measure (defined ‘cone to direct gaze’) narrowing with age until maximum 8–9 degrees (Mareschal et al., 2016). Here, we include faces with a 5 degrees averted gaze as stimulus lying between direct gaze and more explicit gaze and head shifts by 45 and 90 degrees in the Gaze dimension of the search space. Of note, infants’ sensitivity to gaze shifts is typically enhanced by concurrent head turns, so in our paradigm the “averted gaze” faces correspond to head turns by 5, 45 and 90 degrees. Infants’ interest to faces with their head turned away from the infant could be interpreted as an initial response to joint attention in the context of gaze-following tasks (Mundy, 2018). However, according to the Natural Pedagogy theory, emotional faces with their head turned away should not be engaging given the absence of context features that signal their communicative value, such as a gazed-at object and preceding direct gaze (Senju and Csibra, 2008). Thus, we included these stimuli in the search space to test whether the Nc is indeed sensitive to social attention assuming head and eyes away from the infant are the least engaging cues for the infants.

The Nc was obtained in real time after each block presenting one of the possible stimuli in the search space. Subsequently, the Bayesian Optimisation algorithm selected the next stimulus to be presented based on the previous brain responses, with the aim to update the estimated function of the relationship between brain activation and the stimuli in the search space. By leveraging uncertainty of unsampled stimuli and the data points already acquired, the algorithm identified the combination of gaze and emotion information in the parent’s face within the search space that produced the maximal Nc mean amplitude for the tested infant.

**Fig. 2:**
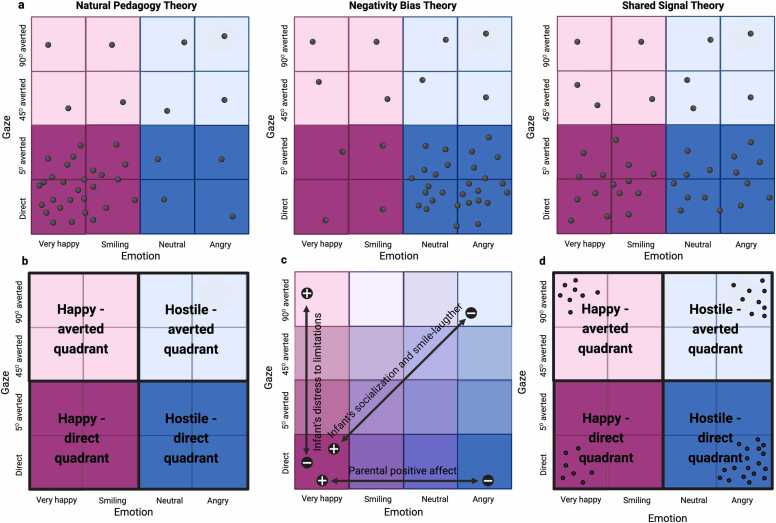
a. Hypothesised pattern of Neuroadaptive Bayesian Optimisation outcome in support of the Natural Pedagogy, Negativity Bias and Shared Signal theories. Each dot represents the hypothesised location of the optimum for one of the tested infants. The number of dots per quadrant in these figures was calculated based on simulations of Bayes test for contingency tables to obtain 98 % probability that the study data support each theory. b. The ‘quadrants’ as referred to in the manuscript. c. Prediction of the relationship between individual differences in infant’s distress to limitations, smile-laughter and socialisation scores and parental positive affect, and the Euclidean distance from the very happy face with direct gaze in the Gaze x Emotion space. + and – signs indicate that according to our prediction, infants whose optimum is closer to the bottom-left corner will show reduced (-) distress to limitations score, and increased (+) socialisation, smile-laughter and parental positive affect scores. d. The results of the Neuroadaptive Bayesian Optimisation experiment. Dots represent the optima for the individual infant that completed the experiment (N=42). Created with BioRender.com.

Our primary goal was to use this individualised neuroimaging-based approach to answer a core question in developmental neuroscience, specifically to distinguish between predictions of competing theoretical explanations of how the infant brain is engaged by faces displaying specific combinations of gaze and emotion cues. We anticipated that NBO would allow us to test the three introduced theories of infant attentional engagement with social cues (Fig. 2.a) by looking at the distribution of individual preferences rather than using a traditional average approach. Our key assumption was that the Nc can be used as a measure of infant attention engagement reflecting social motivation. Accordingly, we expected that for most infants the algorithm would identify the optimal stimulus as the parent’s face with happy expression and direct gaze, as proposed by the Natural Pedagogy theory (Csibra and Gergely, 2009a, Csibra and Gergely, 2009b). Support for this hypothesis would have been provided if the higher proportion of the infants’ optimal stimuli were located within the bottom-left quadrant (the happy-direct quadrant, Fig. 2.b), including very happy and smiling faces with a direct gaze and a 5 degrees averted gaze and head, typically considered direct at this age (Gamer and Hecht, 2007, Mareschal et al., 2016).

However, we acknowledged that NBO results might have supported other explanations of infant’s enhanced engagement with gaze/head direction and emotions in the parent’s face. If in most cases the algorithm predicted the Nc to be enhanced for stimuli located in the bottom-right quadrant of the space illustrated on Fig. 2, representing the parents’ face looking towards the infant without smiling, this would indicate that they engaged most strongly with the cues that are most threatening, as proposed by the Negativity Bias hypothesis (Klucharev and Sams, 2004). The hostile-direct quadrant (Fig. 2.b) includes the parents’ faces with an angry or neutral expression (comparable to the ‘still face’ paradigm that typically distresses young infants, Tronick et al., 1978) and direct or 5 degrees averted gaze, perceived as looking toward the infant. If across the entire sample the algorithm identified the optimum stimulus to be happy (very happy or smiling) or hostile (neutral or angry) faces with a direct (or 5 degrees averted) gaze and head to the same extent, and more than faces with averted gaze, this would indicate that they engage most strongly with the cues that elicit either approach- or avoidance-behaviour, as proposed by the Shared Signal hypothesis (Adams and Kleck, 2003).

Our secondary goal was to use NBO to learn about individual differences in the developmental trajectories of the social brain. We argued that group-level patterns might be overlaid by individual differences, and help us explain variation (deviations from the mean responses in previous group-level findings) within general developmental processes. Therefore, we also related the outcome of the Bayesian Optimisation to infants’ experience and their behavioural characteristics to find out whether they might shape their interest in specific gaze-emotion combinations (Fig. 2.c). We measured parental positive affect using the PANAS (Watson et al., 1988), infant’s social skills with the Socialisation Score of the Vineland Adaptive Behavior Scale (Sparrow et al., 2005) and the Negative Affectivity and Positive Affectivity subscales of the IBQ-Revised (Garstein and Rothbart, 2003). We predicted the optimum stimulus to be further from the very happy face with a direct gaze, and closer to faces with averted gaze and head for infants with higher levels of Negative Affectivity (as suggested by exploratory analyses on an independent dataset using the Distress to limitation IBQ subscale available for the IBQ-Short version, from Gui et al., 2021a, Gui et al., 2021b, see Figure S1). Further, we expected infants with reduced Positive Affectivity scores and social skills in general to be more likely to show preference for non-happy faces with averted gaze and head (Pons et al., 2019), in line with Shultz and colleague’s (2018) suggestion of a circularity between the infant-elicited and the parents-produced social cues. Additionally, we were interested to know whether the interaction between infants’ characteristics and parental positive affect contribute to explain infant’s attention engagement with faces at a neural level (de Haan et al., 2004, Elsabbagh et al., 2014, Pérez-Edgar et al., 2017). If prior experience is what motivates children towards highly communicative cues, we expect the optimum to be closer to the very happy face with a direct gaze for children whose parents report a typically positive mood.

## 2 Materials and methods

### 2.1 Study sample: Eligibility and recruitment

Eligible participants were children aged from 6 months and 0 days to 8 months and 29 days of age. Children were not invited to participate if they had a family or personal history of epilepsy, if they were born before 31 weeks of gestational age, if they had a sensory or motor impairment or any clinical condition. As sources of recruitment, we used the Birkbeck Babylab webpage (http://www.cbcd.bbk.ac.uk/babylab) and social media, and the existing recruitment database at Birkbeck. The database contains information families have provided to us for the purpose of being invited to participate in future research studies. Eligible families were invited by phone or by email to participate in the study.

For the registered report, participants were 53 children (24 females, 29 males) aged from 6 months and 2 days to 9 months and 0 days of age (M age = 7.12 months, SD = 0.95). Recruitment stopped when we obtained a sample size of 40 infants for whom the Bayesian Optimisation algorithm output an optimum value. The final sample for the study includes 42 infants for whom an optimum value was obtained, as two additional families had been already booked in for the experiment when we reached the target sample size and were offered to take part in the study. The target sample size was calculated using G*power version 3.1 (Faul et al., 2009) based on the previous emotion-effect literature; a sample of 38 infants would be needed to obtain a significant difference in Nc mean amplitude between faces displaying happy vs neutral of 1.0 μV (SD=2.4) (Munsters et al., 2019b). Bayes Factor Design Analysis (BFDA, Schönbrodt and Wagenmakers, 2018) with sequential Bayes Factors for minimum 20 and maximum 100 participants indicated that there would have been 100 % probability to obtain supporting evidence (B_10_ > 6) for one tailed paired t-test reporting a difference in the Nc mean amplitude between happy and neutral faces with a Cohen’s d effect of 1.00 (calculated from mean and SD reported in (van den Boomen et al., 2019a, van den Boomen et al., 2019b) with 40 participants, Figure S2). 90 % power would be obtained with N=20, where under the null hypothesis of no difference between happy and neutral faces we would have observed 0.7 % Type-I error results, 53.1 % inconclusive results and 46.2 % true-negative results (BF_01_ < 0.167, Figure S3). Simulation analyses revealed that even if we were to observe effects half the size of what was found by van den Boomen et al., 2019a, van den Boomen et al., 2019b, i.e. a Cohen’s d = 0.5, the alternative hypothesis of a larger Nc to happy than neutral faces would be confirmed in 84 % of the studies with a BF_10_ = 6 with a sample size of 40 infants (see Figure S4).

Of note, these power analyses were based on previous published group effects comparing two of the emotional conditions. This result lays the foundation for the present research but was not its focus. Our primary aim was to test whether the proportion of infants whose optimum corresponds to the parents’ face perceived as happy and directed toward the infant is greater, equal or smaller than the proportion of optima in other portions of the stimuli space. This hypothesis can be tested with a Fisher’s test or Bayesian analysis for contingency tables (see Analyses section), but these tests cannot produce suitable effect sizes for BFDA calculations. Regarding our secondary aim, power analyses conducted with G*power v3.1 (Faul et al., 2009) revealed that 40 children would have also allowed us to detect moderate effect sizes (Cohen’s f^2^>= 0.31, corresponding to R^2^ increase >= 0.088) from the planned multiple linear regression with a power of 80 % (Figure S5).

After conducting the registered analyses, we collected an additional non-registered sample of 21 infants (12 females, 9 males, M age = 7.09 months, SD = 0.72, see ‘Randomised burn-in sample’ column on Table S1) to test whether the order of burn-in images influenced the NBO outcome. The same recruitment criteria and the same experiment were conducted with this sample, except the order of the burn-in images was randomised among participants. The optimum was obtained for 20 of these infants (95 %) as one infant could not complete the experiment due to bad quality data.

### 2.2 Study protocol

Infants and their parent were invited to the Birkbeck Babylab to take part in the EEG real-time experiment. We invited parents independently of their gender, however based on our experience with testing infants in this age range, a lower number of fathers vs mothers were expected to participate. In our registered report, we stated that if our sample included more than five fathers, we would have conducted sensitivity analyses to test whether results were influenced by the participating parent’s gender. Three of the 53 tested participants were accompanied by their father therefore parent’s gender was not included in the analyses as a covariate. While infants previously showed differential ERP (on the N290 component, Righi et al., 2014) and behavioural (Johnson et al., 2021) responses to female vs male faces, potential effects of face gender or familiarity on the Nc can be neglected in the current paradigm, because it studies differences between the expressions and gaze/head directions within the same face instead of between faces. Of note, since the study took place during the day, the parent coming to the lab can be assumed to be the primary caregiver at that time.

The entire protocol was explained in detail prior to the study via e-mail, providing the parents with the opportunity to ask questions. Upon arrival, families were welcomed and situated in a dedicated reception space where the study protocol will be briefly repeated again, and the infant had the chance to familiarise with the experimenters. Parent and infant were shown the EEG cap on a doll or teddy bear to familiarise themselves with the method and get another opportunity to ask questions on the method. Parental consent and assent were obtained for all infants participating in the study.

After consenting to participate, 16 images of the parent’s face displaying different combinations of gaze/head direction and emotional expressions were taken (as in the top box of Fig. 1 and Figure S6). Parents sat comfortably on a chair and the experimenter was in front of them at approximately one metre distance and take the 16 photos using an iPad Wi-Fi 32 GB. To ensure the parents’ pictures were comparable, they were always taken at the same location in the same room, with only artificial lightening on, and with the same wall as background. The iPad was positioned on a tripod located in front of the chair, at a pre-defined distance marked on the floor with a coloured tape. Parents were instructed to remove all accessories except those the children see them with most of the time (for example, glasses).

A sample sheet was provided to parents as an example of the facial expressions and gaze/head directions they were requested to display. Further, the experimenter gave them verbal instructions indicating what emotional expression (“grumpy” for the angry expression stimuli, “neutral” for the neutral expression stimuli, “happy with a small smile” for the smiling stimuli and “happy with a large smile” for the very happy stimuli) and direction for their head and gaze (indicating the parent to look “in front” for the direct gaze stimuli, “toward my ear” for the 5 degrees averted gaze stimuli, “45 degrees to the right, toward the corner of the room” for the 45 degrees averted gaze stimuli, “90 degrees to the right, head and gaze toward the wall on your right” for the 90 degrees averted gaze stimuli). Pictures had a squared frame and white background. Pictures were processed using the *SHINE_color* MATLAB toolbox (Dal Ben, 2019) to control for potential low-level confounds and homogenise the luminance of the 16 pictures. Images were transformed to the CIE Lab colour space and equated in luminance based on histogram matching (see Willenbockel et al., 2010 for details on this method), by iteratively optimising the structural similarity index measure (SSIM). The target luminance histogram was obtained from the source images, using the foreground/background matching with image background specified as white. The mean stimulus luminance of the processed images was saved and used as a covariate in the analyses (see 2.4 Analyses). For seven of the 53 tested participants, the images of the parent’s face could not be processed using foreground/background matching and were presented without being processed. Mean luminance levels to be included in the analyses was calculated *post hoc* using ‘whole image’ matching rather than foreground/background matching. Following image processing, the experimenter took head measurements of the infant and prepare the EEG cap.

For EEG data collection, two experimenters were present, one operating the computer and one helping to guide the infant’s attention to the screen. The infant sat on the parent’s lap approximately 60 cm from a 24-inches screen (1920 ×1200 pxl). The EEG cap was positioned on the child’s head by the experimenter. Subsequently, EEG signal was checked for the 6 frontal channels of interest and two reference channels (see below). During the presentation of the faces, simultaneous video-recording was in place to identify periods when the baby is attentive to the screen using a webcam system installed on top of it and connected to the experimenter’s screen. The experimenter controlled the stimulus presentation by pressing a keyboard key.

EEG data was acquired from the Enobio 8-channels EEG system (Neuroelectrics) and loaded into Matlab using a Lab Streaming Layer (LSL) to connect the EEG recording software to the Matlab software. The EEG data was analysed in real time using a Matlab customised script. An output value for the Nc mean amplitude was saved and used by the optimisation algorithm to select the next stimulus to be presented to the child. The EEG processing and optimisation takes about 5–10 seconds. During this time, a colourful, infant-friendly still-image was presented on the screen. If the infant was fussy, the second experimenter tried to calm the infant by showing them a toy or blowing soap bubbles. The EEG session terminated when a) the optimisation algorithm converged to an optimal stimulus (i.e. after sampling three times the same stimulus), b) the child shows signs of distress, or c) after a maximum of 15 blocks.

#### 2.2.1 EEG paradigm and real-time pre-processing

EEG data was collected using a user-defined 8-channel montage (10–10 EEG coordinate system) including 6 fronto-central electrodes of interest (Fz, Fp1, Fp2, C1, C2 and Cz) and two additional channels for re-referencing during pre-processing (P7 and P8, see Figure S7). CMS and DRL electrodes were placed on the infants’ right mastoid using sticktrodes. Recorded EEG data were referenced online to the CMS channel and digitized at 500 Hz. Before starting the experiment, EEG data quality was visually inspected to make sure a good quality EEG signal was obtained for the majority of the eight channels (Enobio Quality Index > 0.8, where QI depends on power, noise, offset and drift of the signal every two seconds as in Neuroelectrics, 2019).

The entire paradigm consisted of sequential blocks of 12 trials each, with a maximum of 15 blocks. The number of trials was selected to obtain at least 11 valid trials across channels to produce the ERP. The valid trial threshold was calculated based on bootstrapping on a sample of 19 infants presented with images of their mother’s face, as explained in the Supplementary Materials (Figure S8). The maximum number of blocks was defined to avoid the experiment to last more than 20 minutes including breaks (considering ∼12 s for the stimulus presentation, as described below, plus 10 s for the optimisation process per block), since infants start to lose attention to the screen after that amount of time. In each trial, a square face image (1024 ×1024 pxl) centred on the screen appeared on a grey background and remained on the screen for 500 ms. A white cross on a grey background was displayed before each trial for a duration which was randomised between 400 and 600 ms (Xie and Richards, 2016). This serves as a neutral and standardised visual input before the actual stimulus onset. For each block, the same face image was presented, and stimulus presentation was controlled by the experimenter pressing the space bar of the keyboard while the infant looks at the screen. When the key was not pressed, an attention-grabber (red spiral) appeared in the centre of the screen, accompanied by a sound, to redirect the infant’s attention toward the screen. Additionally, the experimenter could encourage the infant to look at the screen by calling his/her name or producing sounds (tapping or using a rattle) behind the screen. Furthermore, a dynamic attention grabber (coloured 1280 ×720 pxl video centred on the screen with the same grey background accompanied by a sound, played for a duration of 2–3 seconds) was presented after trials 4, 7 and 9, in order to facilitate the infants’ attention to the screen, as well as to reduce habituation to the faces.

At the end of the block, EEG data for each block was processed and analysed in real-time using custom-built functions. The EEG data was segmented into 1500 ms-segments around stimulus onset. Each segment was detrended, demeaned, mirror-padded (padding-value: 1000) and band-pass filtered between 0.1 and 20 Hz. It was then cut into segments between 100 ms before and 800 ms after stimulus onset, and baseline corrected (-100 – 0 ms).

Flat channels (signal <0.0001 μV) and channels exceeding a voltage threshold or range during the time window of interest (250–800 ms) were removed. To account for individual differences in the magnitude of the EEG signal, we adopted *default* and *less conservative* criteria: the ERP data of the first block were analysed and visualised using the *less conservative* criteria where artefacts were defined as exceeding a threshold of+/-250 μV amplitude and/or 500 μV range. If the maximum amplitude for that ERP was lower than −200 μV, this less conservative threshold (+/-250 μV) and range +/-500 μV were applied throughout. If the maximum amplitude for that ERP was equal to or higher than −200 μV, the *default* criteria were applied where artefacts are defined as exceeding a threshold of +/- 200 μV or a range of 400 μV. The possibility to apply *less conservative* criteria was used to account for differences in the average ERP amplitude between infants due to different scalp and brain characteristics.

Cleaned EEG data were re-referenced trial-by-trial in order to cancel out ground-related noise and improve signal quality (Neuroelectrics, 2019). To this end, we subtracted the averaged signal of the electrodes P7 and P8 from the signal in the channels of interest. We chose these symmetrical electrodes for re-referencing to ensure this procedure did not cancel out our signal of interest, as would have happened if we would have re-referenced to Cz or other electrodes close to the scalp area where the Nc is recorded. Finally, for each trial, the data was averaged across all samples in all channels of interest.

Given that previous literature was based both on the peak and mean amplitude of the Nc, we operationalised our target brain measure as the mean amplitude of the biggest negative deflection around the negative peak within the classic Nc time window of 250 and 800 ms post-stimulus. If no negative Nc peak was identified for that block, the mean amplitude between 250 and 800 ms was used as target measure for the NBO. This measure (hereafter, Nc negativity) was chosen prior to starting the data collection to capture the neural correlate of infants’ attentional engagement represented by a dipole showing negativity over the central region of the scalp (Gui et al., 2021a, Gui et al., 2021b, Throm et al., submitted). The Nc negativity was calculated across valid trials of the block. The percentage of valid trials across all six frontal channels of interest (6×12 = 72 trials in total) is displayed on the screen. If the percentage of valid trials was equal to or higher than 16 %, corresponding to 11 valid trials (similar to previous Nc research, Gui et al., 2021a, Gui et al., 2021b; Munsters et al., 2019b; Nelson and de Haan, 1996, see also Figure S8) the ERP output value was saved and received as input by the Bayesian Optimisation algorithm. If the percentage of valid trials was below the 16 % threshold, the block was repeated.

Of note, the proposed pre-processing pipeline has been initially created and tested in a proof-of-principle study (da Costa et al., 2021) and subsequently piloted in 9 more infants. The entire pipeline, EEG testing Standard Operating Procedures and scripts are available online *[view-only for peer review at this stage]*: https://osf.io/8yfv2/?view_only=c341e03e7838489f820c059b3a5bd632.

The traditional Nc mean amplitude (i.e., mean of all the samples between 250 and 800 ms) and the mean amplitude of the event-related brain activity between 0 and 200 ms post-stimulus was analysed offline at the end of the entire data collection, applying the same pre-processing scripts to EEG data obtained from the six fronto-central channels.

#### 2.2.2 Optimisation

The NBO approach in infant EEG has been illustrated in detail in (da Costa et al., 2021). The approach relies on a Bayesian Optimisation algorithm that efficiently finds extrema of unknown functions, f(x)=yby fitting a statistical model to the sampled values and determining where to sample next based on the previously obtained values and their uncertainty (Brochu et al., 2010). By balancing between exploration and exploitation, the algorithm will find the function extrema (representing the stimuli that produce the largest Nc negativity, in the present case) in a small number of blocks. Bayesian Optimisation is composed of two main parts: the surrogate model and the acquisition function. The surrogate model, a statistical model of the unknown objective function, f(x), uses a Gaussian process regressor to build the statistical model based on previously sampled values, GP(x)=p(x|y) (Rasmussen and Williams, 2006). The covariance of the predicted function is specified by a kernel, whose hyperparameters are optimised during the fitting of the model by maximising the log-marginal-likelihood. We used the stationary Matern kernel with a smoothness parameter υ = 2.5 and an added white noise term that estimated the global noise level of the data. For every block of the Bayesian Optimisation, the prediction of f(x) and its standard deviation were passed to the acquisition function.

In the present study, the Bayesian Optimisation algorithm aimed to optimise the Nc negativity by targeting its most negative value (reflecting enhanced attention engagement, Richards et al., 2010). In the first 4 iterations, or ‘burn-ins’ to Bayesian Optimisation, the presented stimuli were pre-defined as the algorithm needed to capture an initial model of the Nc negativity’s variation across the experimental search space. The number of burn-in images was chosen as previous work in adults indicated four burn-ins allowed us to obtain a sufficiently robust starting point for subsequent optimisation (Lorenz et al., 2016, Lorenz et al., 2021). In the present experiment, the four initial points sampled lied at the corners of the search space, corresponding to: angry with direct gaze (Angry-0), angry with 90 degrees averted gaze and head (Angry-90), very happy with direct gaze and head (VeryHappy-0), very happy with 90 degrees averted gaze and head (VeryHappy-90). In the registered study, the order of the burn-in stimuli was kept constant across participants to avoid image presentation order to be a possible reason for inter-individual differences in the NBO outcome. Following completion of the registered analyses, the burn-in stimuli presentation was randomised for an additional (non-registered) sample to verify whether the order of burn-in stimuli influenced the NBO outcome.

In the real-time paradigm, the stimulus was presented to the infant and the Nc negativity was obtained after automatic processing as explained above. Following the burn-in phase, the system ran iteratively in a closed-loop and the acquisition function defined which stimulus to display next. Based on previous pilot work (see (da Costa et al., 2021, Throm et al., 2023), the Bayesian Optimisation parameter ξ value was set to 0.1, which benefited exploitation of identified maxima that allowed us to minimise the number of blocks needed to reach the optimal solution, corresponding to the stimulus that reliably produced the larger Nc negativity. Thus, the acquisition function progressively chose a stimulus to sample that was estimated to be closer to the predicted maximum until the same image was chosen three times consecutively, indicating that algorithm had converged to the unknown function’s maximum. This was assumed to correspond to the optimal stimulus that produced the stronger brain activation. In case the optimal solution was not found, we planned for the paradigm to stop after 15 iterations, corresponding to 15 blocks. At the end of the paradigm, when the algorithm found the optimum, the NBO output the position on the search space estimated to produce the strongest negativity of the Nc for the tested child. These coordinates on the space were used in the analyses to identify the optimal stimulus for that child and to compute the Euclidean distance between the optimal stimulus and the predicted optimum (VeryHappy-0). Figure S9 displays the ERP and Bayesian Optimisation outputs by block for one pilot participant (male, age 6 months 28 days).

### 2.3 Behavioural measures

#### 2.3.1 Infant questionnaires

Parents were provided with a unique URL with access to three questionnaires about themselves and the child. Responses were directly entered into the Gorilla online research tool (https://gorilla.sc/, Anwyl-Irvine et al., 2020).

The IBQ-R is a widely-used parent-report measure developed to assess dimensions of temperament in infants between 3 months and one year of age (Garstein and Rothbart, 2003). This tool allows to reliably measure individual differences in reactivity and self-regulation that complement laboratory assessments (Bosquet Enlow et al., 2016). The IBQ-R Very Short form was administered to parents in form of an online questionnaire on a Likert scale ranging from 1 (“Never”) to 7 (“Always”) plus a “Does not apply” option, on 37 questions regarding infant’s behaviour in daily situations such as “When being dressed or undressed during the last week, how often did the baby squirm and/or try to roll away?” or “During a peekaboo game, how often did the baby laugh?”. As mentioned, the Smile-laughter subscale score of the IBQ was used in previous research looking at Nc peak amplitude in response to emotional faces (de Haan et al., 2004). Further, preliminary analyses showed that enhanced Nc mean amplitude, indicating attention engagement, to averted vs direct gaze was associated to increased Distress to limitation in the dataset from Gui et al., 2021a, Gui et al., 2021b (illustrated on Figure S1). Because these scales cannot be computed from the Very Short form of the IBQ that was administered in this study, the Negative Affectivity and the Positive Affectivity scales, that included some items of the Distress to limitations and Smile and laughter subscales, respectively, were used in this study to reflect infant’s negative or positive emotional expressions that might influence experience with parental cues as proposed by Shultz et al. (2018).

The Vineland Adaptive Behavior Scales (VABS) is a semi-structured interview measuring adaptive functioning in everyday life (Sparrow et al., 2005). It has been extensively used to capture variability in adaptive behaviour and differences in developmental trajectories in infants and toddlers (Estes et al., 2015, Bussu et al., 2018). In the present study, VABS was collected as a parent-report online questionnaire. Parents were asked to answer whether their child’s shows specific behaviours in their daily life with one of with five options (“Usually”, “Sometimes or Partially”, “Never”, “Don’t know” or “No opportunity”). We were interested in responses to the VABS Social Skills and Relationship subscale scores, which includes questions such as “Looks at face of parent or caregiver” or “Smiles or makes sounds when approached by a familiar person”.

#### 2.3.2 Parental questionnaire

The Positive And Negative Affect Scale (PANAS, Watson et al., 1988) consists of 10 positive and 10 negative words that describe feelings and emotions the parent might have experienced in the past months. Parents were asked to respond based on a 5-items Likert scale indicating how often they generally experience the listed feelings (from “Very slightly or not at all” to “Extremely”). Total positive and negative scores can be created from this questionnaire. The parental positive score was found to interact with infants’ Smile-laughter score in affecting the Nc peak amplitude to emotional faces by de Haan et al., (2004), and was used in the present research.

### 2.4 Analyses

#### 2.4.1 Neuroadaptive Bayesian optimisation to confirm theories of social attention

Planned analyses and hypotheses are described in Table 1 and illustrated in Fig. 2. To test whether the NBO approach supported the Natural Pedagogy theory of selective attention engagement to face over the Shared Signal and Negativity Bias theories, we investigated whether the proportion of children for whom the Bayesian Optimisation algorithm selected the socially engaging faces, displaying positive emotional expressions and directed toward the infant (bottom-left quadrant in Fig. 2.b) was greater than, equal to or less than the proportion of children whose optimal stimulus was a face directed toward the infant with a hostile expression (bottom-right quadrant in Fig. 2.b). Infants were excluded from this analysis if the session was terminated before the algorithm has converged (e.g., because the infant became fussy). The average number of blocks required for convergence in the entire sample is reported as a result and discussed in light of the proposed advantages of the NBO approach in infant research (Gui et al., 2022).

**Table 1:** Planned analyses in the registered report. ‘Quadrants’ are represented on Fig. 2.b.

Question	Test	Key variables	Covariates	Expected direction
Do Neuroadaptive Optimisation results support the natural pedagogy framework of preferential attention engagement to faces?	Bayesian analysis	-Proportion of infants with optima in the happy-direct quadrant -Proportion of infants with optima in the hostile-direct quadrant -Proportion of infants with optima in the happy-averted quadrant -Proportion of infants with optima in the hostile-direct quadrant	/	Natural Pedagogy: Proportion in the happy-direct quadrant > proportion in the hostile-direct quadrant Negativity Bias: Proportion in the happy-direct quadrant < proportion in the hostile-direct quadrant Shared Signal: Proportion in the happy-direct quadrant = proportion in the hostile-direct quadrant and their sum > proportion in hostile-direct + happy-averted quadrants
Does a classic design approach with the present experimental data support the natural pedagogy framework of preferential attention engagement to faces?	Two (gaze)-by-two (emotion) ANOVA	Dependent variable: Nc mean amplitude in the four ‘burn-in’ blocks (very happy / direct gaze, very happy / 90° averted, angry / direct gaze, angry / 90° averted)	-Age in months -Mean proportion of trials -Mean stimulus luminance [-sex in sensitivity analyses]	Significant effect of gaze (larger Nc to direct vs 90° averted gaze) Significant gaze- emotion interaction (larger Nc to 90° averted gaze / direct gaze for very happy faces only)
Which combination of gaze and emotion on a face is the Nc most sensitive to?	One-tailed t-test against chance level	Proportion of infants with optima in each of the 16 possible stimuli in the search space	/	Proportion > 6.25 % with Bayes Factor > 3
Does infant’s preferential attention at the neural level reflect individual differences in infant behavioural characteristics and their interaction with parental mood?	Hierarchical multiple linear regression	Dependent variable: Euclidean distance from very happy / direct gaze Independent variables: Baseline model: covariates only Model 1: Baseline + Negative affectivity score + Positive affectivity score + Socialisation standard score Model 2: Model 1 + Parental positive affect score Model 3: Model 2 + Parental positive affect score * Negative affectivity score Model 4: Model 3 + Parental positive affect score * Positive affectivity score Model 5: Model 4 + Parental positive affect score * Socialisation standard score Model 6: Model 5 + child’s sex	-Age in months -Mean proportion of trials -Mean stimulus luminance -Child’s sex	Positive relationship between Euclidean distance from very happy / direct gaze and Negative affectivity score Negative relationships between Euclidean distance from very happy / direct gaze and Positive affectivity, Socialisation scores and parental positive affect Significant increase in R^2^ for Model 1 and 4.

We performed a Bayesian analysis (using the *BayesFactor* package in R) to estimate whether the proportion of infants with optima located in the happy-direct quadrant of the search space (including very happy and smiling faces with direct gaze and 5 degrees averted gaze and head, see Fig. 2.b) was higher, lower or equal to than those in the hostile-direct quadrant (including neutral and angry faces with direct gaze and 5 degrees averted gaze and head). If the parent’s face was selected as optimum by the NBO when infants perceive it as happy and directed towards them more than when it is perceived as hostile, this would indicate that the Nc reflects infants’ attention engagement with highly communicative cues on the parent’s face, in line with the Natural Pedagogy theory.

A priori simulations using Bayes test for contingency tables revealed that this result was 6 times more likely to occur (considered as ‘compelling evidence’, Schönbrodt and Wagenmakers, 2018) if at least 27 of the 40 infants have their optimum in the bottom-left quadrant (happy-direct, see Fig. 2.b) and maximum 5 infants have their optimum in the bottom-right quadrant (hostile-averted), with a simulated posterior probability difference > 0.60 (Figure S10.a). If the proportion of optimal stimuli located in the hostile-direct quadrant was higher than that in the happy-direct quadrant with a difference >0.66 (Figure S10.b), this would indicate that the Nc reflects mechanisms of attention engagement that are more in line with the Negativity Bias theory.

If the proportion difference between happy-direct and hostile-direct is between −0.31 and 0.32 (Figure S10.c), and the proportion of infants with optimum on the bottom-half of the space (direct and 5 degrees averted gaze) is higher than the proportion of infants with optimum over the top-half of the space (45 and 90 degrees averted gaze), with minimum 28 of the 40 infants having their optimum for a face perceived to look at them directly, results would be more likely to support the Shared Signal theory with a Bayes Factor = 6.17 (see Fig. 2.a).

To further explore the space and identify what stimulus was preferred above chance level amongst all, we performed a Bayesian proportion test for each of the stimuli identified as optima testing whether proportions were greater than chance level, defined as 1/16=0.063. Bayes factors of 3 or above would be considered as positively supporting the alternative hypothesis that specific stimuli were preferentially selected by most infants, forming further evidence for or against the theories. *A priori* Bayesian analyses revealed that if 8 individuals have their optimum on one stimulus, this would be considered strong evidence that the stimulus has been chosen above chance (BF_10_=10.76).

In the ‘Randomised burn-in sample’, a non-registered one-tailed chi-square test was conducted to test whether the proportion of infants for whom the optimum corresponded to the first burn-in stimulus was greater than what would have been observed if the optimum corresponded to the first burn-in stimulus by chance (0.25, as there were four possible burn-in stimuli).

#### 2.4.2 Classic ERP design

To test whether our results were consistent with the previous literature using a classic ERP design, we performed a 2×2 ANOVA using only the ERP data collected in the burn-in phase. We tested whether there was a Gaze (direct vs 90 degrees averted gaze and head) x Emotion (very happy vs angry) effect on the traditional Nc mean amplitude, controlling for age in months, mean stimulus luminance and mean proportion of valid trials across the burn-in blocks per infant as covariates. Infants were excluded from the analysis if they did not complete the first four blocks.

Based on the behavioural and ERP literature described above, we hypothesised an effect of Gaze with larger Nc to direct vs averted gaze (Gui et al., 2021a, Gui et al., 2021b). Additionally, we expected a significant interaction where a larger Nc mean amplitude is seen to the parent’s very happy face with direct vs 90 degrees averted gaze and head, but no difference due to gaze/head direction in the angry Emotion condition (Doi et al., 2010, Rigato et al., 2010). Of note, these exploratory analyses included all infants with valid EEG data obtained during the burn-in phase, independently on whether the Bayesian Optimisation algorithm converged, as only data from the burn-in phase were used.

As a control, we calculated offline the mean amplitude of the event-related brain activity recorded from the six fronto-central channels between 0 and 200 ms post-stimulus for the four burn-in blocks. We tested whether it was affected by gaze/head direction and emotional expression in a 2×2 ANOVA. Age in months, mean stimulus luminance and mean proportion of valid trials across the burn-in blocks per infant were included as covariates. We expected no effect of gaze and emotion on the signal on the early time-window, since it occurs earlier than the cognitive process we were interested in.

#### 2.4.3 Relationship with infant and parental behaviour

Our secondary goal was to explore whether the output of this individualised method reflected individual differences in behavioural characteristics and parental positive affect. A hierarchical regression model was used to test the association between distance from very happy face with direct gaze and the behavioural measures of negative affectivity and positive affectivity scores measured with the IBQ-R, VABS Socialisation standard score and parental positive affect measured with the PANAS.

The dependent variable was the Euclidean distance in the bi-dimensional search space between the infant’s optimal stimulus and the very happy face with direct gaze. In the baseline model, age in months, mean stimulus luminance and the mean proportion of valid trials across all blocks per infant were the only independent variables. In Model 1, we added to this baseline model the three infant behaviour scores. The parental positive affect variable was included as a predictor in Model 2 and, subsequently, the interaction of parental positive affect with infants’ negative affectivity (Model 3), positive affectivity (Model 4) and socialisation (Model 5) added progressively (as indicated in Table 1). Last, the child’s sex was added as additional covariates in Model 6.

Significant changes of the model’s R^2^ were examined using F statistics to test whether they contributed to explain variability in the infant’s optimal stimulus distance from very happy face with direct gaze. We expected a significant increase in R^2^ in Model 1, with a positive relationship between distance from very happy face with direct gaze and infant negative affectivity (see Figure S2) and negative relationships between distance from very happy face with direct gaze and infant’s positive affectivity and socialisation scores (Pons et al., 2019). A positive coefficient for the relationship between distance and parental positive affect was also expected in Model 2 (Elsabbagh et al., 2014, Pérez-Edgar et al., 2017). Based on de Haan et al. (2004), we predicted a significant interaction between infant’s negative affectivity and parental positive mood would be seen in Model 3, and a significant interaction between infant’s positive affectivity and parental positive mood would be seen in Model 4 (see Fig. 2.c).

To further investigate significant findings, in the model with the lowest Bayesian Information Criterion (BIC) only, we explored whether the Gaze or Emotion dimension were linked to the behavioural measures. In multiple logistic regressions, we tested whether the behavioural measure significantly associated with optimal stimulus distance in the previous analysis was associated with distance across the x and y axis of the experimental search space.

## 3 Results

Fifty-three infants (24 females and 29 males) and their parents took part in the present experiment. The infants’ parent-defined ethnicity is reported on Supplementary Table S1; 34 (64 %) were defined as white, 12 (23 %) of mixed ethnicity and for 7 (13 %) parents did not reply to the ethnicity question. For five infants the experiment was interrupted by the experimenter due to infants’ fussiness and excessive movement. For six additional infants, experimental issues prevented us to acquire the data (N=3 due to MATLAB crashing when dealing with excessively noisy data, N=2 due to errors in the image processing step, and N=1 to problems with the EEG data acquisition). Therefore, forty-two infants completed the experiment, corresponding to 79 % of the tested infants (17 females, 25 males, M age = 7.02 months, SD = 0.95). Of these, one reached the 15 blocks stopping criteria, while for 41 infants, the algorithm converged within 8.68 blocks on average (SD = 2.09, min = 6, max = 14).

Our primary goal for the present study was to test whether the Neuroadaptive Bayesian Optimisation results supported one of the main theories of infant attentional engagement towards combinations of gaze/head direction and emotional facial expressions. As registered, the 42 infants who completed the experiment were entered in this analysis. Fig. 2.d shows the distribution of optima for the 42 individual infants across the 16 stimuli in the search space. The stimuli that were predicted to elicit the strongest Nc negativity were: the angry face with direct gaze for 18 infants (Angry-0 = 43 %), the angry face with 90 degrees averted gaze and head for 9 infants (Angry-90 = 21 %), the very happy face with direct gaze for 7 infants (VeryHappy-0 = 17 %), and the very happy face with 90 degrees averted gaze and head for 8 infants (VeryHappy-90 = 19 %).

As planned, we performed a Bayesian analysis to test whether the proportion of children for whom the algorithm converged on the bottom-left quadrant of the stimulus space illustrated on Fig. 2.b (happy facial expressions with direct gaze) was higher, in support of the Natural Pedagogy theory, or lower, in support for the Negativity Bias theory, than the proportion of children with a maximum in the bottom-right quadrant (hostile facial expressions with direct gaze). We found positive evidence (Bayes Factor = 3.24, Trujillo-Barreto, 2015) that the proportion of optima in the hostile-direct quadrant was higher than the proportion in the happy-direct quadrant, in line with the Negativity Bias theory. There was no support for the Shared Signal theory, as the evidence that the two proportions are equal was BF = 0.31. Additionally, the hypothesis that the images with direct gaze (bottom half of the stimulus space in Fig. 2.b) were more likely to elicit a stronger Nc negativity than the images with averted gaze and head (top half of the stimulus space) was not supported (BF = 0.69).

When examining stimuli individually, we found evidence that the Bayesian Optimisation algorithm identified the four corners of the stimulus space, which were also the four stimuli presented as burn-ins (Angry-0, Angry-90, VeryHappy-0, VeryHappy-90) to elicit the strongest brain response for more infants than would have been expected by chance (1/16=0.063, see Table 2 for the BF). All but the VeryHappy-0 image were selected by more than 8 individuals, number indicated by *a priori* analyses as indicator of strong evidence. After observing that the algorithm identified the optimum among the four corners of the stimulus space only, we conducted additional, non-registered analyses to check whether stimuli were identified against chance level defined as 1/4 corners =0.25. We found positive evidence that only Angry-0 (BF = 5.33) was identified as optimum more often than would have happened by chance (see Table 2 for all the other BFs < 3).

**Table 2:** Information about the Neuroadaptive Bayesian Optimisation results (column 1), Bayesian theory testing (columns 2 and 3) and mean event-related component calculations (columns 4, 5, 6) for the four stimuli at the corners of the stimulus search space: angry face with direct gaze (Angry-0), angry face with 90 degrees averted gaze and head (Angry-90), very happy face with direct gaze (VeryHappy-0), very happy face with 90 degrees averted gaze and head (VeryHappy-90). Of note, for the Nc mean amplitude, more negative values correspond to stronger brain activation. Conversely, for the Nc negativity values, more positive values correspond to higher negativity therefore stronger brain activation.

	Number (percentage) of infants for whom it was identified as optimum (N=42)	Bayes factor for test for selection against chance (1/16)	Bayes factor for test for selection against chance (1/4)	Mean (SD) Nc mean amplitude in burn-in phase (N=50)	Mean (SD) Nc negativity in burn-in phase (N=50)	Mean (SD) early ERP mean amplitude in burn-in phase (N=50)
Angry-0	18 (43 %)	103799350	5.33	-6.46 (9.27)	3.45 (10.1)	-1.09 (5.25)
Angry-90	9 (21 %)	23.64	0.47	-8.05 (8.91)	3.73 (8.27)	-0.78 (6.11)
VeryHappy-0	7 (17 %)	3.42	0.86	-9.70 (11.3)	7.53 (11.9)	-2.03 (7.97)
VeryHappy-90	8 (19 %)	8.31	0.60	-9.25 (7.20)	6.10 (8.10)	-1.74 (4.47)

The same pattern of results was observed when including the non-registered ‘Randomised burn-in sample’ (N=62, see Supplementary Note). In the ‘Randomised burn-in’ sample, the Bayesian Optimisation algorithm converged on the first burn-in image for 25.0 % of the infants (N=5), which is not more often than expected by chance (χ2(1)=0, p=0.5).

**Fig. 3:**
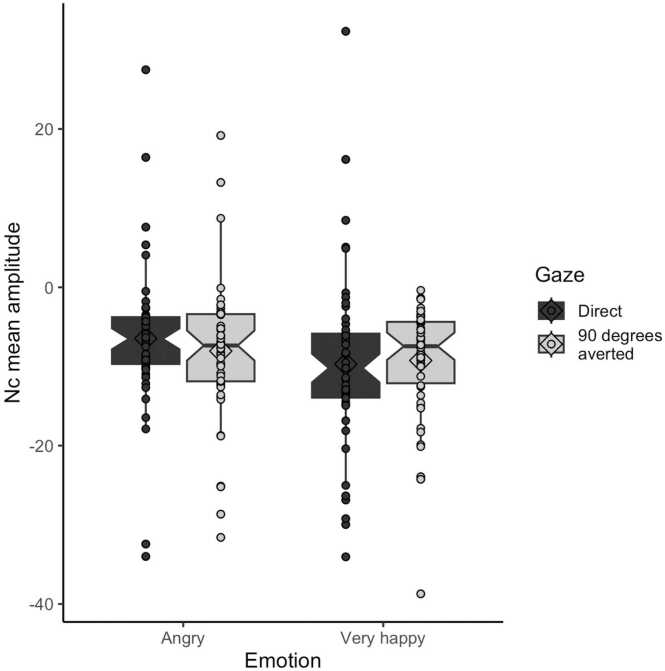
Classic event-related potential results illustrating mean Nc amplitudes (in micro-Volts, on the y axis) by emotional expression (on the x axis) and gaze/head direction (dark grey = direct, light grey = 90 degrees averted). Dots correspond to individual infants’ mean Nc amplitude for each of the four corners of the stimulus space.

We next performed classic ERP analyses using the data for the burn-ins (i.e., the first four, pre-defined images that were presented) to test whether our data analysed with traditional, inferential group-level approaches would be in line with previous findings. For the purpose of comparability with previous group-level findings, we calculated the Nc mean amplitude across the entire traditional time window of 250–800 ms. For this analysis, we included all individuals who contributed to the first four blocks, independently of whether they completed the experiment, as pre-registered (N = 50, from the originally recruited children we excluded 1 child for whom EEG data was not acquired, and 2 children for whom we had no MATLAB file with EEG data). Contrary to our expectations, we found no significant effect of Gaze on the Nc mean amplitude (F(1,46) = 0.24, p = 0.620, generalised η^2^ = 0.001) nor emotion (F(1,46) = 3.69, p = 0.061, η^2^_G_ = 0.015), although an overall larger Nc was observed to very happy vs angry faces (see Table 2 and Fig. 3). The interaction between Gaze and Emotion was also not significant (F(1,46) = 0.79, p = 0.378, η^2^_G_ = 0.003). All the covariates’ effects and interactions between covariates and within-subject variables were non-significant (*p*s < 0.084). Adding the child’s sex as a covariate did not change the pattern of results (see Supplementary Table S2 for the results). Supplementary Figure S11 shows the same pattern of results when using the Nc negativity (our target measure for the NBO) as dependent variable.

We also calculated offline the mean amplitude of the ERP between 0 and 200 ms post-stimulus, which was not expected to be influenced by Gaze and Emotion. Repeated measures ANOVA confirmed that, in line with our predictions, the early ERP was not influenced by Gaze (F(1,45) = 0.08, p = 0.783, η^2^_G_ < 0.001), Emotion (F(1,45) = 1.27, p = 0.266, η^2^_G_ = 0.008) or the interaction of the two (F(1,45) = 0.011, p = 0.918, η^2^_G_ < 0.001). All *p*s were non-significant (> 0.061) except for the interaction between mean proportion of valid trials and Gaze (F(1,45) = 5.13, p = 0.028, η^2^_G_ = 0.035).

To check that NBO results were indeed capturing meaningful signal, we ran an additional analysis that was not pre-registered. We sub-grouped the children based on the location of their optima in the stimulus space and tested whether in each subgroup the Nc negativity was higher for the condition corresponding to children’s optimum. Results (reported in the Supplementary Figure S12) confirmed this and validated the optimum identified by the Bayesian algorithm as reflecting the predicted stimulus that produced the most enhanced brain activation rather than a random choice.

**Fig. 4:**
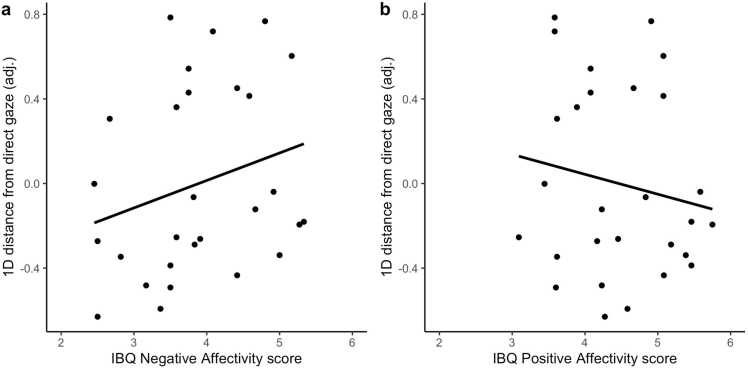
Relationship between distance from the very happy face with direct gaze along the Gaze dimension and infant Negative (**a**) and Positive (**b**) Affectivity scores of the Infant Behavior Questionnaire (x-axes). Distance (y-axes) has been adjusted by regressing out the effects of experimental covariates (infant’s age, mean proportion of valid trials and mean luminance), socialisation scores and parental positive affect included in the logistic model.

In hierarchical regression analyses, we investigated whether the Euclidean distance in the bi-dimensional search space between the infant’s optimal stimulus and the very happy face with direct gaze was significantly associated with the infant’s negative affectivity, positive affectivity and socialisation (Model 1), parental positive affect (Model 2) and the interaction between infant and parent’s behavioural measures (Models 3, 4 and 5). This analysis was conducted on the 28 individuals for whom parents completed all the questionnaires. Contrary to our expectations, behavioural measures did not significantly improve prediction of the NBO results (all *p*s > 0.208, see Table S3). In the model with the lowest BIC including all predictors (Model 2), we observed a positive relationship between distance and infant’s negative affectivity, although it was not statistically significant (β = 0.56, SE = 0.29, p = 0.063, Fig. 4), suggesting that children who were more engaged with the face with direct gaze showed lower levels of negative affectivity. The direction of association between distance and infant’s positive affectivity was also in line with the expectations, but non-significant (β = −0.14, SE = 0.36, p =0.686). However, we also reported mild positive relationships between distance and infant’s socialisation (β = 0.02, SE = 0.02, p = 0.686) and parent’s positive affect (β = 0.08, SE = 0.06, p = 0.208), contrary to the predictions. None of the covariates had effects on the optimum distance from the very happy-direct corner (all *p*s < 0.515).

Although the hierarchical regression yielded no significant result, we explored our one-dimension hypotheses (illustrated on Fig. 2.c) using multiple logistic regressions including the same variables that were in Model 2 as predictors, and distance across the Gaze or Emotion dimension, respectively, as dependent variables. Along the Gaze dimension, we observed a significant positive association with infant’s negativity score (β = 5.05, SE = 2.27, p =0.026), a significant negative association with infant’s positivity score (β = −4.26, SE = 1.94, p =0.027) and a significant positive association with parents’ positive affect (β = 0.64, SE = 0.32, p =0.046). This indicates that infants whose optimum was a face with averted gaze and head had more negative and less positive affectivity, and parents with more positive affect. The relationship with socialisation scores was non-significant (β = 0.04, SE = 0.09, p =0.639).

There were no significant associations between behavioural measures and distance along the Emotion dimension (Table S4).

The hierarchical regression analysis conducted on a combined sample of N=45 infants (28 from the registered sample and 17 from the ‘Randomised burn-in’ sample) confirmed that behavioural measures did not significantly improve prediction of the NBO results (Table S5).

## 4 Discussion

In this registered report, we applied Neuroadaptive Bayesian Optimisation to identify the combination of emotion and gaze cues in the parent’s face that could elicit the strongest attentional engagement at the brain level in individual 6- to 9-month-olds. Our primary aim was to use the NBO results to test theories about preferential attention to specific social cues. In line with the Natural Pedagogy theory, we expected the algorithm to identify the image of the parent’s very happy face with direct gaze as the stimulus that was most likely to produce the strongest brain correlate of attention engagement (a negative ERP over the fronto-central regions of the scalp between 250 and 800 ms after stimulus onset, or Nc negativity). However, our results were not in line with this hypothesis and instead supported the Negativity Bias theory, according to which most infants should be most engaged when seeing the parent’s face displaying an angry facial expression and looking directly at them. While this was observed for the largest group of children with a converging algorithm (43 %), NBO still allowed us to collect information about the preferences of the children who did not show the most common response. Our secondary aim was to test whether the NBO outcome was linked to infant’s affect and social behaviour in everyday life, with their experience with the parent’s emotional state, and with the interaction of the two. We did not find evidence that these behavioural measures significantly explained the distribution of the optima in the stimuli search space. However, we observed trends in the data indicating that infants whose brain activation was enhanced in response to faces with averted gaze/head had higher negative and lower positive affectivity at a behavioural level, in line with our predictions. This secondary result is encouraging with respect to the possibility that NBO might be a useful tool to capture individual differences in brain correlates of behaviour during early development. Overall, this paper provided evidence that the NBO approach could be used to test whether theories elaborated to explain group-level findings apply to individual-level results. Further, it demonstrated that an individualised experimental approach has the potential to refine our understanding on general developmental processes.

### 4.1 Evaluation of the method

The NBO results allowed us to test whether most of the children were more likely to exhibit a stronger brain activation in response to specific combinations of emotional expressions and gaze/head direction. The NBO and the classic ERP approach produce findings on different levels, with the former producing individual-level optima based on infant-selected stimuli and the latter producing group-level responses to pre-selected stimuli. The fact that NBO identified the Angry-0 face as the stimulus that elicited the strongest Nc in the largest group of children, while the classic ERP analysis on the burn-ins showed a strongest Nc for the VeryHappy-0 face overall could raise concern that the NBO outcome was selected at random. To demonstrate this was not the case, we conducted a non-pre-registered analysis testing whether individual optima truly reflected the stimulus that was predicted to produce the strongest Nc in the infants. We sub-grouped infants based on their optimum and were able to verify that across all blocks the optimal stimulus was the one that elicited the most negative Nc (Supplementary Figure S11). The NBO results are not likely to be driven by low-level image characteristics, as there were no significant differences between the four stimuli in the amplitude of an early ERP calculated between 0 and 200 ms after the stimulus presentation. Additionally, no significant effects of the number of valid trials or mean luminance were found in the classic ERP analysis. The order of images presentation in the burn-in phase was also not likely to have influenced the NBO result given that, at the group level, strongest Nc amplitudes were observed for VeryHappy-0 rather than for the most frequent optimum (Angry-0). To rule out this possibility, we tested an additional sample of 20 infants where we randomised the order of the burn-in images. The data confirmed that the first image presented to the infants was not selected as optimum by the algorithm more than what was expected by chance. Last, it could be argued that differences between classic ERP and NBO results are due to the fact that the Nc negativity, corresponding to the mean amplitude of the negative ERP around the Nc peak, rather than Nc mean amplitude was used in the NBO. We chose this measure because the literature indicated both peak amplitude and mean amplitude as being modulated by gaze and emotion which are combined in this measure, and because it has previously proved to be a robust metric of attention engagement on the single-block level in the individual infant (Throm et al., under review; preregistered https://doi.org/10.17605/OSF.IO/CWF96). For the NBO to work effectively, the target brain measure needs to be a reliable correlate of the cognitive function that the algorithm is aiming to optimise. The pattern of results we obtained in the classic ERP analysis with Nc mean amplitude as dependent variable was largely observed also when replacing it with the Nc negativity (see Table 2 and Figure S11).

### 4.2 Theoretical implications

The literature brings forward several accounts as to why infants find some cues in social interaction more interesting than others. The NBO approach allows us to test multiple hypotheses within the same study and thus can help disentangle evidence for different theories. With the classic ERP approach comparing mean Nc amplitude values by Gaze and Emotion (examined in the burn-in phase of the experiment, when stimuli presentation was pre-defined as in traditional designs rather than being guided by the infant’s responses), we found no significant preference at the group level. On the contrary, the fact that individual optima were identified in virtually all infants completing the study shows that infants do have attentional preferences for specific combination of social cues but that these preferences are heterogeneous. This offers an explanation for the inconsistent findings reported in the introduction.

Comparing proportions of individual optima (rather than averaged brain responses) yielded support for the Negativity Bias theory that states that infants are more engaged by environmental stimuli that carry a negative valence, compared to a positive one (Vaish et al., 2008). Attending more to negative stimuli carries a greater advantage from an evolutionary point of view, as it allows animals and humans to prioritise keeping safe from potentially harmful situations (Rozin and Royzman, 2001). Increasing evidence from the field of psychology and neuroscience suggests that the Negativity Bias is justified by the observation that equivalent negative and positive inputs do not produce equivalent outputs. According to the Evaluative Space Model, higher levels of negative inputs tend to produce stronger outputs than higher levels of positive outputs, and this leads the cognitive system to prioritise them (Norris, 2021). For example, learning is quicker and more effective in the context of negative reinforcement compared to positive reinforcement, as the aversion conditioning mechanism can even bypass cognition (Garcia et al., 1974). Allocating greater attentional resources to the parents’ angry face with direct gaze might be an adaptive behaviour justified by the need to avoid aversive situations (Nelson et al., 1979). Further, it might be that frequent exposure to parental positive expressions in the first six months of age might make the negative expression more salient especially for the Nc (Vaish et al., 2008), which is known to be influenced by novelty (Richards, 2008). This explanation of the NBO results would also explain the unexpected positive association between the individual infants’ optimum distance from the VeryHappy-0 image in the search space and parental positive affect. This indicated that infants whose parents tended to display fewer positive emotions were more likely to attend preferentially to the VeryHappy-0 stimulus, perhaps due to its relative novelty. Of note, given that these results are not statistically significant, these should be considered speculative observations that will require further investigations. The sensitivity of the Nc to novelty might also explain the results if parents found it challenging to perform a “grumpy” expression as indicated and produced a facial expression that was ambiguous to the infants, increasing their attention levels.

While the NBO approach showed support for the Negativity Bias theory, when brain responses were averaged across the first four presented blocks and across infants in the classic ERP analysis, we obtained non-significant results with a trend (albeit non-significant) towards stronger Nc amplitudes for VeryHappy-0 was observed (Fig. 4), in line with predictions of the Natural Pedagogy theory. Traditionally, group-level results would be interpreted as reflective of a general developmental process, although when looking at the individual datapoints inter-individual differences are evident. Thus, the data confirm that classic experimental designs can be limited when it comes to draw conclusions about theories that are highly affected by inter-individual variability (Almaatouq et al., 2022, Haartsen et al., 2024). Taken together, the classic ERP and NBO results support the idea that attentional preferences are heterogeneous across the space and highlight the importance of including individual-/subgroup-level measures in study designs.

### 4.3 Relation between preferred stimuli and infant characteristics

A unique strength of the NBO approach over using traditional group-level neuroimaging designs is that it outputs a prediction of the individual infant’s preferences. While current results yielded support to the Negativity Bias theory proposing most infants to preferentially attend to Angry-0, the remaining individual optima were distributed across the stimulus space. We asked if the heterogeneously distributed individual optima associated with infant characteristics. While overall infants’ and parental behavioural characteristics did not contribute to explain the distribution of the optima within the stimulus space, exploratory analyses revealed that the NBO outcome along the Gaze dimension of the space was associated with infants’ affectivity, in line with our predictions. Specifically, we observed that infants whose optimum was a face with direct gaze presented less negative affectivity (i.e., more distress, crying and clinginess) and more positive affectivity (i.e., laughs and excitement), as reported by their parents. On the contrary, infant’s and parent’s affectivity was not significantly related to attentional preference to emotional expressions as identified by the NBO. One possible explanation for the negative finding of the relationship between the infants’ responses on the combined Gaze x Emotion stimulus space and their characteristics is that our dependent variable, namely the “Euclidean distance from VeryHappy-0 in the stimulus space”, is suboptimal for capturing how the Nc maps the interaction between the two social cues. In other words, as discussed above in light of the Evaluating Space Model (Norris, 2021), attending preferentially to the Angry-0 face does not have the same “value” of attending to the VeryHappy-90 face as our Euclidean distance measure indicated. In fact, while the Euclidean distance metric treats variability across the two dimension of the space as essentially the same, they might be qualitatively different. Indeed, our exploratory one-dimensional analyses testing the relationship of behavioural variables with the Gaze and Emotion separately seem to support this idea. As we relied on parents’ emotional expressions to provide infants with a range of emotional expressions that infants would normally experience in everyday life, we let parents dose for themselves how “grumpy” their expression would be. However, it could also be that for some parents the “grumpy” expressions did not show the same intensity as the “very happy” expressions. Additionally, the fact that only four of the 16 possible stimuli in the space were chosen as optima possibly limited the possibility to capture nuanced associations between brain and behaviour.

### 4.4 Limitations and future directions

This study proposed a novel approach with a registered plan for analyses and provided robust evidence for the use of real-time EEG in combination with artificial intelligence taking a transformative approach to evaluate theories in developmental psychology and neuroscience (Haartsen et al., 2024). However, it has several limitations that need to be addressed in future research employing the NBO approach. First, the Bayesian Optimisation algorithm almost exclusively sampled the initial burn-in images (corners of the search space) rather than fully exploring the search space. This is partly because we prioritised exploitation in the algorithm parameter definition, in order to effectively obtain an output within the minimum number of blocks. Developing approaches that allow a longer exploration period while maintaining infant attention will be fruitful, this study will allow us to optimise the acquisition function to better balance exploration and exploration going forward. The present study is a registered report and therefore all the methodological choices were taken before starting data collection, exclusively based on previous papers and pilot work. Future studies will build on the results of this study and inform adjusting the algorithm parameters and implementation to improve the NBO method with infant’s neuroimaging data. Additionally, more work should be done to systematise some aspects of the search space, such as the intensity of the parents’ facial expressions, while preserving the individualised nature of the paradigm in personalised experiments.

Second, despite prioritising exploitation for rapid identification of the optimum, the attrition rate (21 %) in this study is only slightly lower than in classic ERP studies (25 %, van der Velde and Junge, 2020). This is somewhat disappointing given that we expected that this adaptive paradigm could be more engaging and lead to lower data loss. In fact, the attrition due to infant fussiness (11 %) was much lower than typically observed with traditional paradigms (21–23 %, van der Velde and Junge, 2020). However, the complexity of the paradigm (including the collection and processing of the 16 pictures of the parent before running the experiment) and high computational demand for the EEG pre-processing and Bayesian Optimisation scripts to be performed in tandem and in real time were responsible for almost half of the data loss (5 infants of the 11 excluded, 11 % vs 2–4 % reported in classic ERP studies). Future NBO studies need to take these aspects into consideration when designing the experiment. Incorporating this kind of technique into commercial packages would increase robustness of the system and possibly simplify the analysis pipeline. This would allow us to add an eye-tracking component and make the paradigm gaze-contingent, so that it will be paced by the infant itself.

Third, while the target sample size was reached, as per the registered report, for the primary aim, the total sample for the secondary aim was much lower (N=28 vs N=42 in the primary analysis) due to missing questionnaire data (N=6, 14 %) or because they answered “Don’t know” to more than two items in at least one of the subdomains (Interpersonal Relationships or Play/Leisure), which makes the subdomain invalid and consequently the overall socialisation domain score invalid too (N=8, 19 %). While we acknowledge that analyses might be underpowered to reveal significant results, we also note that a larger sample (N=45) obtained combining the original sample with the non-registered sample tested with randomised burn-in stimuli did not yield significant results either (Table S5). This suggests that if a relationship between the optima position in the search space and the tested infants and parents’ behavioural characteristics exists, its effect is quite small and might not meaningfully contribute to explain individual differences in the development of social cognition. Fourth, for seven infants, the images could not be processed and homogenised based on background luminance prior to the testing session. Mean luminance was calculated post-hoc and included in the analyses. Adding post-hoc luminance adjustment as a dummy covariate in the classic ERP and hierarchical regression analyses did not change the pattern of results.

In conclusion, we showed the potential of applying real-time EEG data collection combined with AI-based methods in young infants to simultaneously test different theories on the infant brain’s engagement with specific combinations of gaze and emotion cues when looking at faces. We showed that most infants were likely to present a stronger Nc negativity when attending to angry faces with direct gaze, in line with the Negativity Bias theory. The NBO approach produced individual level results that were partly explained by infant’s individual differences in behaviour. Specifically, children who tended to be more engaged towards faces with direct gaze presented higher positive affectivity and lower negative affectivity according to parental report. These initial findings are encouraging towards new applications of NBO to study individual differences in neurodevelopment and social cognition.

## Funding

AG and RH were supported for this study by the 10.13039/501100000269Economic and Social Research Council grant n. ES/R009368/1 to EJHJ and RL. ET, PFDC, FP and MAM were supported by the European Union’s HORIZON 2020 Research and Innovation Programme under the Marie Sklodowska-Curie Grant Agreement No 814302. AJB was funded through the iCASE studentship as part of the UCL-Birkbeck Medical Research Council (10.13039/501100000265MRC) Doctoral Training Partnership (DTP) grant (MR/W006774/1) to EJHJ and AG. This study is also supported by EU-AIMS (European Autism Interventions), which received support from the Innovative Medicines Initiative Joint Undertaking under grant agreement no. 115300, the resources of which are composed of financial contributions from the European Union’s Seventh Framework Programme (grant FP7/2007–2013), from the European Federation of Pharmaceutical Industries and Associations companies’ in-kind contributions, and from Autism Speaks as well as AIMS-2-TRIALS which received support from the Innovative Medicines Initiative 2 Joint Undertaking under grant agreement No 777394. This joint undertaking receives support from the European Union’s Horizon 2020 research and innovation programme and EFPIA and AUTISM SPEAKS, Autistica, SFARI (RH, EJHJ). Disclaimer: The views expressed are those of the authors and not necessarily of the IMI 2JU.

## Ethical Statement

The study has received ethical approval by the Birkbeck Departmental Ethics Committee of the Department of Psychological Science (Ref. No. 192001).

## Declaration of Competing Interest

The authors declare that they have no known competing financial interests or personal relationships that could have appeared to influence the work reported in this paper

## Data Availability

The data have been uploaded on UK Data Archive and is currently under review (ReShare code 856957)
