# Supplementary material for: Neuroadaptive Bayesian optimisation to study individual differences in infants’ engagement with social cues
Source: Dev Cogn Neurosci. 2024 Jun 10;68:101401. doi: 10.1016/j.dcn.2024.101401 (PMC11225696; doi:10.1016/j.dcn.2024.101401)
Supplement: Supplementary file 1 — Supplementary material [file mmc1.docx]

**Supplementary Figures**


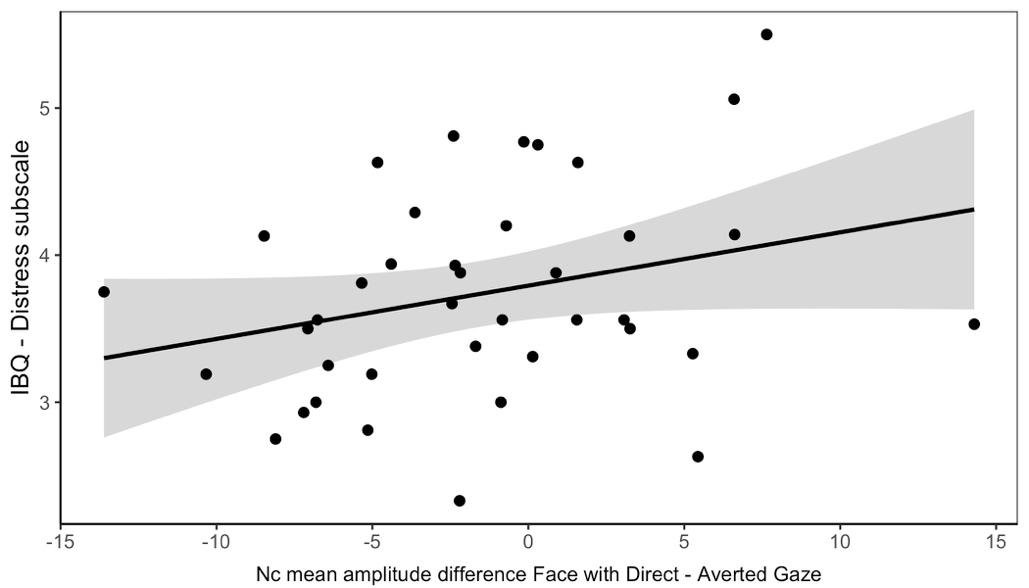


*Figure S1*. Preliminary analysis of the relationship between Nc mean amplitude difference between Faces with Direct vs Averted Gaze and the Infant Behavior Questionnaire (IBQ) – Distress to Limitations subscale in the typically developing infants from Gui et al., (2021).


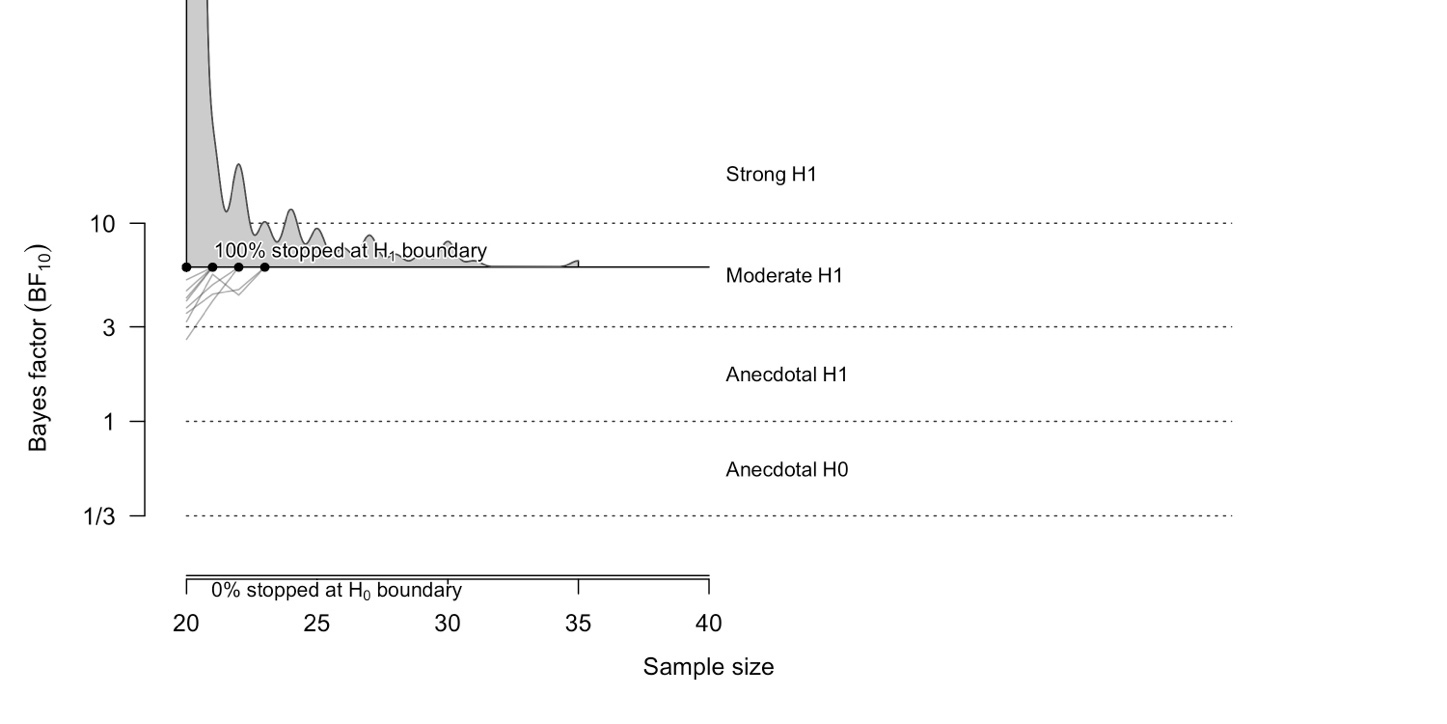


*Figure S2*. Sequential Bayes Factor Design Analysis (Schönbrodt & Wagenmakers, 2018) with a sample size of 40, illustrating the probability that results support the alternative hypothesis of higher Nc mean amplitude in response to happy faces compared to neutral faces with a Cohen’s d = 1.0, calculated based on estimates from (van den Boomen et al., 2019)with BF_10_=6, BF_01_=1/6.


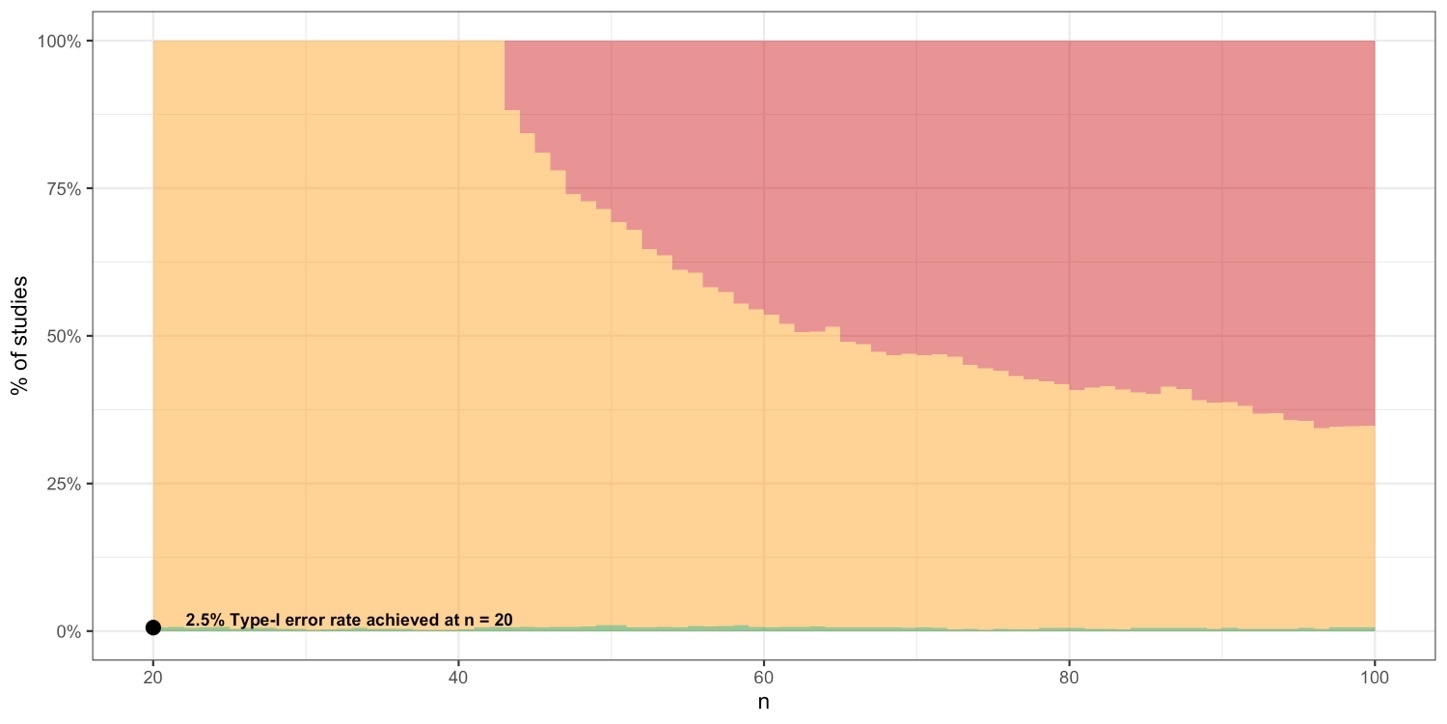


Figure S3. Bayes Factor Design Analysis (Schönbrodt & Wagenmakers, 2018): Expected probability of Type I error based on sample size for higher Nc mean amplitude in response to happy faces compared to neutral faces with a Cohen’s d = 1.0, calculated based on estimates from (van den Boomen et al., 2019).


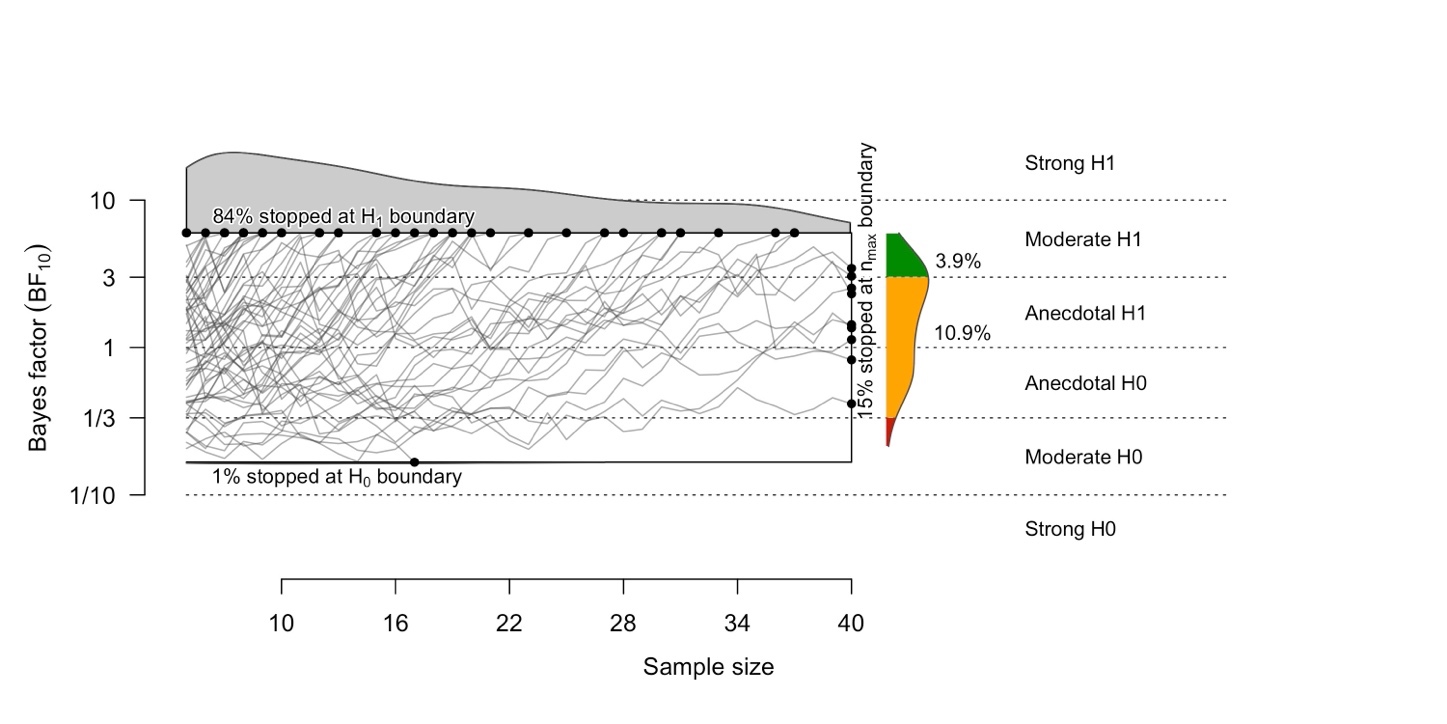


*Figure S4*. Sequential Bayes Factor Design Analysis (Schönbrodt & Wagenmakers, 2018) with a sample size of 40, illustrating the probability that results support the alternative hypothesis of higher Nc mean amplitude in response to happy faces compared to neutral faces with a Cohen’s d = 0.5 with BF_10_=6, BF_01_=1/6.

*Figure S5*. Effect size detectable with 80% power as a function of the sample size for a linear multiple regression model with three predictors of interest and two additional covariates (see Table1, Model 1), calculated with G*Power v3.1 (Faul et al., 2009). The sample size for the present study is 40.

*Figure S6*. Example of the Gaze (y-axis) x Emotion (x-axis) search space, with images equated in luminance.


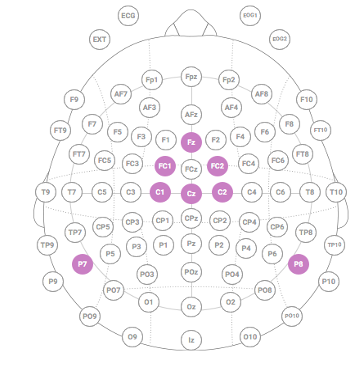


*Figure S7*. Eight-channel montage used in the present study.


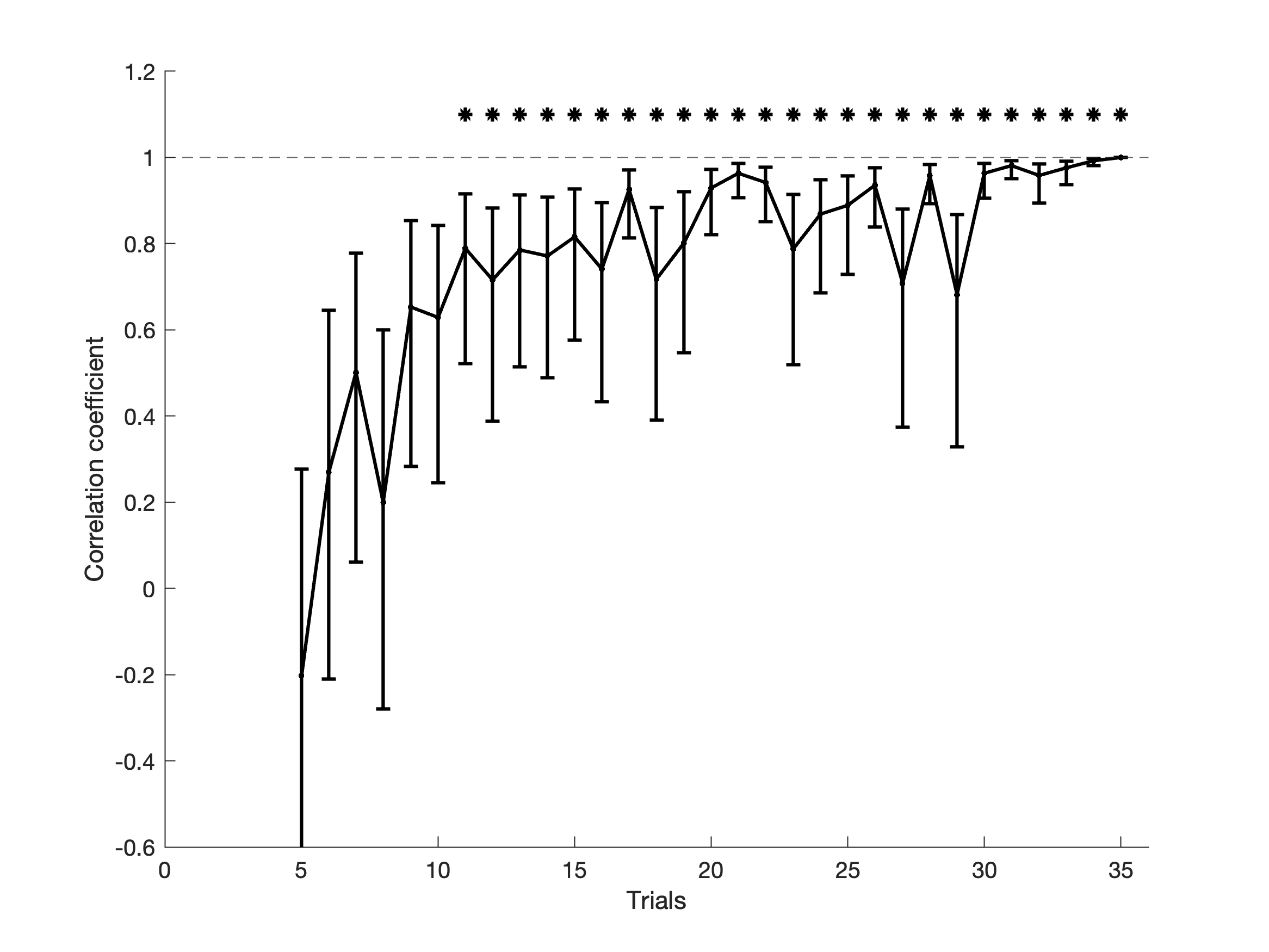


*Figure S8.* Bootstrapped Nc mean amplitude correlation across trials.

Bootstrapping analyses were conducted on an existing dataset of 19 infants aged 6 to 12 months from a similar ongoing study (DOI: [10.17605/OSF.IO/CWF96](https://doi.org/10.17605/OSF.IO/CWF96)). EEG data was collected in response to images of the mother’s face with direct gaze and neutral facial expression for minimum 35 valid trials (collected in four blocks). For each infant participant, the distribution of the Nc mean amplitude for N=5 to 35 trials was computed by randomly selecting the N trials 1000 times for each infant and calculating the Nc mean amplitude for that infant. We then tested the correlation between the bootstrapped Nc mean amplitudes for N=5 to 34 trials and that obtained with N=35 trials (assumed to be the ‘true’ Nc) across the entire sample. The figure shows the correlation coefficients by trial, with the upper and lower 95% confidence intervals as error bars. Asterisks indicate whether the correlation test was significant at a Bonferroni-corrected p-value = 0.05/30 tests = 0.002.

*Figure S9*. Pilot Data: ERP and Bayesian Optimization output of a pilot infant participant (male, age 6 months and 28 days. For each block, the Mean Amplitude of the Nc is computed and the percentage of valid trials by channel included in the ERP is displayed. The stimuli space (Component 1 = Emotion, Component 2 = Gaze) is colour coded on based on the Nc negativity value predicted by the Gaussian Process (yellow for more enhanced, i.e., more negative, Nc) and variance of the prediction is produced by the Bayesian Optimisation algorithm after taking into account the Nc value for that block. For this pilot infant, the optimum stimulus is localised on coordinates (1, -1), corresponding to the parent’s angry face with direct gaze.

*Figure S10*. Distribution of the posterior probability simulated over 10,000 repetitions under the Natural pedagogy (a), Negativity Bias (b) and Shared Signal (c) hypotheses.


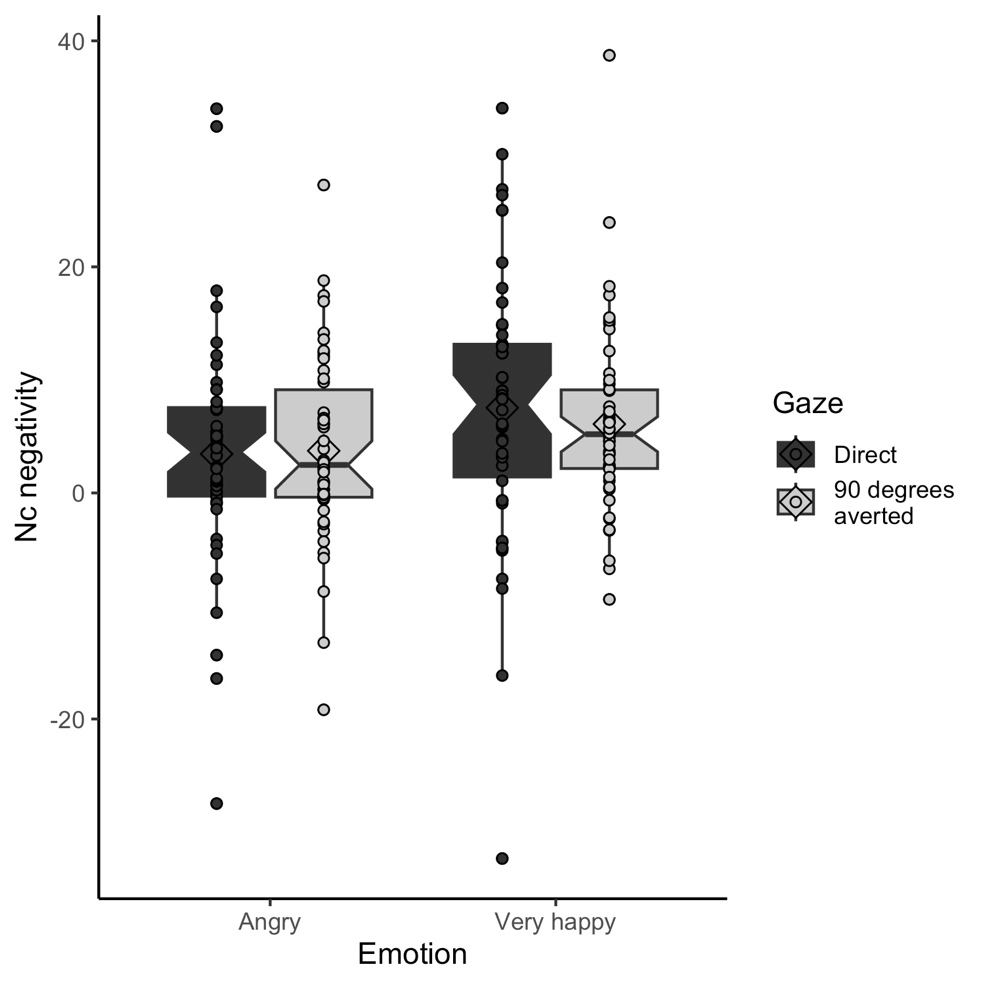


*Figure S11*. Nc negativity (in micro-Volts, on the y axis, with higher values indicating stronger brain activation) by emotional expression (on the x axis) and gaze direction (dark grey = direct, light grey = 90 degrees averted). Dots correspond to individual infants’ mean Nc amplitude for each of the four corners of the stimulus search space. Of note, more positive Nc negativity values correspond to higher negativity therefore stronger brain activation.

In non pre-registered analyses, we checked whether the Nc negativity measure that we used as target measure for the Neuroadaptive Bayesian Optimisation gave similar results (N = 50). Indeed, we observed the same pattern, with a significant effect of emotion (F(1,46) = 6.57, p = 0.014, η^2^_G_ = 0.029), due to stronger Nc negativities for the very happy (M = 6.82, SD = 10.20) vs angry faces (M = 3.59, SD = 9.16). No other effect was statistically significant in this analysis (ps > 0.130), except the interaction between the children’s age and gaze (F(1,46) = 4.06, p = 0.050, η^2^_G_ = 0.015), with significantly stronger Nc negativity for older children in the averted gaze conditions (β = 1.83, SE = 0.87, p = 0.038) and a non-significant negative association in the direct gaze condition (β = -0.46, SE = 1.21, p = 0.704). This confirmed that the Nc negativity reflected attentional engagement as expected based on previous studies, and was possibly a more sensitive measure of the neural correlate we intended to map with the NBO.


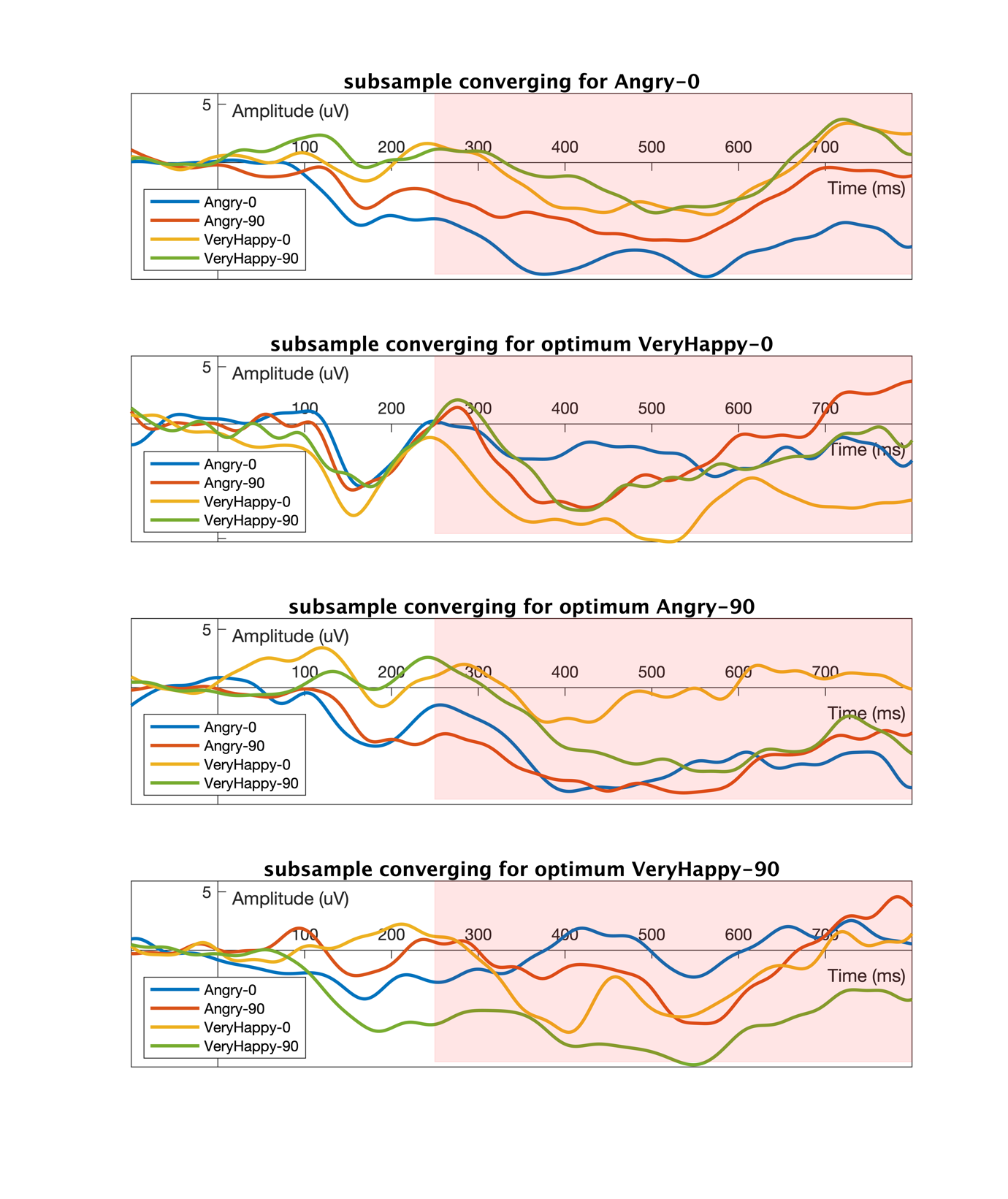


*Figure S12*. Grand averages of the Nc waveform across the experiment, by optimum subgroups. In each of the four optimum subgroups, repeated-measures ANOVA of Nc negativity revealed a significant effect of condition (Angry-0: p < 0.0001, η_p_^2^ = 0.34; VeryHappy-0: p = 0.001, η_p_^2^ = 0.58; Angry-90: p = 0.02, η_p_^2^ = 0.33; VeryHappy-90: p = 0.012, η_p_^2^ = 0.4), with the average Nc negativity being highest for Angry-0 in the Angry-0-optimum-subgroup, for VeryHappy-0 in the VeryHappy-0-optimum-subgroup, for Angry-90 in the Angry-90-optimum-subgroup, and for VeryHappy-90 in the VeryHappy-90-optimum-subgroup.

**Supplementary Tables**

*Table S1*. Parent-reported ethnicity for the infants who participated in the study.

| ethnicity | N initial sample | N RANDOMISED BURN-IN SAMPLE |
| --- | --- | --- |
| Black or Black British - Caribbean | 0 | 1 |
| Mixed - white and Asian | 5 | 3 |
| Mixed - white and black African | 2 | 0 |
| Mixed - white and black Caribbean | 1 | 1 |
| Mixed - any other mixed background | 4 | 2 |
| White - British | 25 | 9 |
| White - Irish | 1 | 1 |
| White - any other white background | 8 | 3 |
| Did not reply | 7 | 1 |

*Table S2*. Results of the repeated measures ANOVA analysis with the inclusion of sex as a covariate.

|  | DFn | DFd | F | p-value | η^2^_G_ |
| --- | --- | --- | --- | --- | --- |
| Mean luminance | 1 | 43 | 0.045 | 0.832 | 4.70E-04 |
| Proportion of valid trails (burn-in) | 1 | 43 | 0.005 | 0.943 | 5.27E-05 |
| Infant’s age | 1 | 43 | 0.878 | 0.354 | 9.00E-03 |
| Infant’s sex | 1 | 43 | 2.012 | 0.163 | 2.00E-02 |
| Gaze | 1 | 43 | 0.029 | 0.866 | 1.14E-04 |
| Emotion | 1 | 43 | 2.388 | 0.130 | 1.00E-02 |
| Mean luminance x gaze | 1 | 43 | 0.011 | 0.917 | 4.35E-05 |
| Prop valid trials x gaze | 1 | 43 | 3.014 | 0.090 | 1.20E-02 |
| Infant’s age x gaze | 1 | 43 | 1.904 | 0.175 | 7.00E-03 |
| Infant’s sex x gaze | 1 | 43 | 0.05 | 0.824 | 1.98E-04 |
| Mean luminance x emotion | 1 | 43 | 1.413 | 0.241 | 6.00E-03 |
| Prop valid trials x emotion | 1 | 43 | 1.298 | 0.261 | 5.00E-03 |
| Infant’s age x emotion | 1 | 43 | 0.689 | 0.411 | 3.00E-03 |
| Infant’s sex x emotion | 1 | 43 | 0.087 | 0.769 | 3.59E-04 |
| Gaze x emotion | 1 | 43 | 0.489 | 0.488 | 2.00E-03 |
| Mean luminance x gaze x emotion | 1 | 43 | 1.322 | 0.257 | 6.00E-03 |
| Prop valid trials x gaze x emotion | 1 | 43 | 2.912 | 0.095 | 1.40E-02 |
| Infant’s age x gaze x emotion | 1 | 43 | 2.331 | 0.134 | 1.10E-02 |
| Infant’s sex x gaze x emotion | 1 | 43 | 0.228 | 0.635 | 1.00E-03 |

DFn = degrees of freedom numerator, DFd = degrees of freedom denominator, η^2^_G_ = generalised eta squared.

*Table S3*. Results of the model fit comparison testing the relationship between optimum distance from the very happy face with direct gaze and behavioural measures.

|  | Res.DF | RSS | DF | SS | F | p-value | BIC |
| --- | --- | --- | --- | --- | --- | --- | --- |
| Baseline | 24 | 21.739 |  |  |  |  |  |
| Model 1 | 21 | 17.519 | 3 | 4.220 | 1.611 | 0.226 | 92.988 |
| Model 2 | 20 | 16.148 | 1 | 1.370 | 1.569 | 0.228 | 94.040 |
| Model 3 | 19 | 15.996 | 1 | 0.153 | 0.175 | 0.681 | 97.106 |
| Model 4 | 18 | 15.812 | 1 | 0.183 | 0.210 | 0.653 | 100.115 |
| Model 5 | 17 | 15.477 | 1 | 0.336 | 0.384 | 0.544 | 102.847 |
| Model 6 | 16 | 13.972 | 1 | 1.505 | 1.723 | 0.208 | 103.315 |

Res.DF = residuals degrees of freedom, RSS = residuals sum of squares, DF = model comparison’s degrees of freedom, SS = sum of squares, BIC = Bayesian Information Criterion.

*Table S4.* Results of the multivariable logistic regression testing the relationship between optimum distance along the Emotion dimension and the infant and parent’s behavioural measures, controlling for the effect of infant’s age, mean proportion of valid trials and mean image luminance.

|  | ß | SE | z | p-value |
| --- | --- | --- | --- | --- |
| (Intercept) | -17.421 | 9.699 | -1.796 | 0.073 |
| Infant’s negative affectivity | -0.295 | 0.852 | -0.346 | 0.730 |
| Infant’s positive affectivity | 1.658 | 1.219 | 1.360 | 0.174 |
| Parent’s positive affect | -0.036 | 0.181 | -0.202 | 0.840 |
| Infant’s age | -0.724 | 0.573 | -1.264 | 0.206 |
| Mean prop valid trials | -5.354 | 4.898 | -1.093 | 0.274 |
| Mean luminance | 0.125 | 0.065 | 1.935 | 0.053 |

SE = standard errors

*Table S5*. Results of the model fit comparison testing the relationship between optimum distance from the very happy face with direct gaze and behavioural measures in the combined sample of 28 infants from the original sample and 17 infants from the non-registered ‘Randomised burn-in’ sample (total N = 45).

|  | Res.DF | RSS | DF | SS | F | p-value | BIC |
| --- | --- | --- | --- | --- | --- | --- | --- |
| Baseline | 39 | 36.487 |  |  |  |  |  |
| Model 1 | 36 | 33.388 | 3 | 3.098 | 1.050 | 0.384 | 146.781 |
| Model 2 | 35 | 31.947 | 1 | 1.441 | 1.466 | 0.235 | 148.624 |
| Model 3 | 34 | 31.421 | 1 | 0.526 | 0.535 | 0.470 | 151.678 |
| Model 4 | 33 | 31.302 | 1 | 0.119 | 0.121 | 0.730 | 155.295 |
| Model 5 | 32 | 31.297 | 1 | 0.006 | 0.006 | 0.939 | 159.071 |
| Model 6 | 31 | 30.482 | 1 | 0.814 | 0.828 | 0.370 | 161.695 |

Res.DF = residuals degrees of freedom, RSS = residuals sum of squares, DF = model comparison’s degrees of freedom, SS = sum of squares, BIC = Bayesian Information Criterion.

**Supplementary Note**

The additional ‘Randomised burn-in’ sample of 21 infants, the algorithm converged within 6 to 14 blocks (M number of blocks = 8.89, SD = 2.37) for 18 infants. For two infants, 15 blocks were reached, and the Bayesian optimisation algorithm calculated the optimum based on the obtained data. For one infant, the experiment was interrupted as the data quality was low for three consecutive blocks.

Of the 21 infants in the ‘Randomised burn-in’ sample, 17 had also available questionnaire data. For two infants parents did not complete the questionnaires and two infants parents answered “Don’t know” to two or more items that contribute to the VABS Socialisation scale, making the datasets invalid for calculation of the socialisation standard scores.

The optimum corresponded to the Angry-0 quadrant of the search space for 30% of the infants (for 5 infants the optimum was the angry face with direct gaze and for 1 infant it was the neutral face with direct gaze), Angry-90 for 45% of the infants (9 infants all had their optimum as the angry face with averted gaze and head), VeryHappy-0 for 15% of the infants (the very happy face with direct gaze was the optimum for 3 infants) and VeryHappy-90 for 15% of the infants (2 infants’ optimum was the very happy face with averted gaze and head for 2 infants). In this sample, there was evidence that the angry face with direct gaze (BF = 5.39) and the angry face with averted face and head (BF = 3188) were selected as optima more often than what expected by chance (all the other BFs < 1.09).

Adding this sample to the initial registered sample produced the same pattern of results as observed before. The data supported the Negativity Bias theory (BF = 4.73) as the overall proportion of optima located in the Angry-0 quadrant (39%) was higher than the proportion of optima in the Happy-0 quadrant (0.16). The Shared Signal theory predicting that the proportion of optima with direct gaze is not higher than the proportion of optima with averted gaze and head was not supported (BF = 0.39).

REFERENCES

Faul, F., Erdfelder, E., Buchner, A., & Lang, A. G. (2009). Statistical power analyses using G*Power 3.1: Tests for correlation and regression analyses. *Behavior Research Methods*, *41*(4), 1149–1160. https://doi.org/10.3758/BRM.41.4.1149

Gui, A., Bussu, G., Tye, C., Elsabbagh, M., Pasco, G., Charman, T., Johnson, M. H., & Jones, E. J. H. (2021). Attentive brain states in infants with and without later Autism. *Translational Psychiatry*, *11*(196).

Schönbrodt, F. D., & Wagenmakers, E. J. (2018). Bayes factor design analysis: Planning for compelling evidence. *Psychonomic Bulletin and Review*, *25*(1), 128–142. https://doi.org/10.3758/s13423-017-1230-y

van den Boomen, C., Munsters, N. M., & Kemner, C. (2019). Emotion processing in the infant brain: The importance of local information. *Neuropsychologia*, *126*(September 2017), 62–68. https://doi.org/10.1016/j.neuropsychologia.2017.09.006
